# Convergence and Divergence in the Evolution of the APOBEC3G-Vif Interaction Reveal Ancient Origins of Simian Immunodeficiency Viruses

**DOI:** 10.1371/journal.ppat.1003135

**Published:** 2013-01-24

**Authors:** Alex A. Compton, Michael Emerman

**Affiliations:** 1 Molecular and Cellular Biology Graduate Program, University of Washington, Seattle, Washington, United States of America; 2 Divisions of Human Biology and Basic Sciences, Fred Hutchinson Cancer Research Center, Seattle, Washington, United States of America; University of Pennsylvania School of Medicine, United States of America

## Abstract

Naturally circulating lentiviruses are abundant in African primate species today, yet their origins and history of transmitting between hosts remain obscure. As a means to better understand the age of primate lentiviruses, we analyzed primate genomes for signatures of lentivirus-driven evolution. Specifically, we studied the adaptive evolution of host restriction factor *APOBEC3G* (*A3G*) in Old World Monkey (OWM) species. We find recurrent mutation of *A3G* in multiple primate lineages at sites that determine susceptibility to antagonism by the lentiviral accessory protein Vif. Using a broad panel of SIV Vif isolates, we demonstrate that natural variation in OWM *A3G* confers resistance to Vif-mediated degradation, suggesting that adaptive variants of the host factor were selected upon exposure to pathogenic lentiviruses at least 5–6 million years ago (MYA). Furthermore, in members of the divergent *Colobinae* subfamily of OWM, a multi-residue insertion event in *A3G* that arose at least 12 MYA blocks the activity of Vif, suggesting an even more ancient origin of SIV. Moreover, analysis of the lentiviruses associated with *Colobinae* monkeys reveal that the interface of the A3G-Vif interaction has shifted and given rise to a second genetic conflict. Our analysis of virus-driven evolution describes an ancient yet ongoing genetic conflict between simian primates and lentiviruses on a million-year time scale.

## Author Summary

The emergence of AIDS in the late 20^th^ century has provoked studies to better understand the evolutionary history of viruses and the factors that govern their spread. Pandemic human immunodeficiency virus-type 1 (HIV-1), which currently infects 34 million people worldwide, emerged following the transmission of a lentivirus between chimpanzees and humans. A growing list of apparently nonpathogenic, species-specific strains has now been characterized in dozens of African primates, suggesting that primate lentiviruses are older and more widespread than originally thought. To estimate the extent to which primates and lentiviruses have coexisted, we examined the interaction between host and virus on a molecular level and tracked its dynamics over evolutionary time. We report that the immunity factor *APOBEC3G* is evolving in tandem with the lentiviral accessory gene *vif*, allowing us to associate instances of host evolution with instances of lentivirus infection in deep and shallow timescales. Specifically, we show that the region of APOBEC3G targeted by Vif is adaptively diversifying in independent primate lineages in a manner that suggests that lentiviruses are millions of years old. Our study reveals that, while primate lentiviruses may have modern consequences for human health, they have ancient origins in our non-human primate relatives.

## Introduction

HIV-1 was introduced into human populations in the early 20^th^ century following multiple transmissions of a chimpanzee virus, known as SIVcpz [1], [2]. The other, less virulent human lentivirus, HIV-2, resulted from transmissions of SIVsm, a virus found in sooty mangabeys [3]. In fact, more than 40 non-human primate species in sub-Saharan Africa are infected with species-specific strains of SIV [4]. Known as “natural hosts,” these species either co-evolved with their respective lentivirus or were infected more recently via cross-species transmission from other primates [5]. In either case, the association between natural hosts and SIV is thought to be considerably older than that of humans and HIV [6], [7]. Natural SIV infections do not generally cause an AIDS-like immunodeficiency in their autologous host species, leading to the hypothesis that the virus-host relationship has evolved towards an apathogenic state [8]–[11]. However, the age and pathogenic potential of wild SIV infections in diverse primate taxa remain largely uncharacterized.

Initial attempts at calculating the age of SIV using phylogenetics produced widely disparate, but all relatively recent, estimates [12], [13]. However, two significant findings have pushed back considerably the age estimates of primate lentiviruses. First, the discovery of a full-length endogenous SIV in the genomes of lemurs indicates that lentiviruses were present in prosimians at least 4 million years ago (MYA) [14], [15]. However, SIV is not currently found in prosimians, while it is common in simian primates like OWM. Thus, the age of lentiviral infections in current natural hosts of SIV cannot be addressed by endogenous lentiviruses.

Second, a date to calibrate SIV phylogenetics in OWM was made possible with the identification of SIV strains endemic to the African island of Bioko. Here, each virus found on the island shares ancestry with a mainland virus, and their respective hosts belong to the same genus, demonstrating that lentiviruses have been infecting OWM for tens of thousands of years [16]. Nonetheless, the use of viral sequences to establish the age of virus families is problematic because rapid evolution obscures phylogenetic signals, and because many viral lineages have gone extinct in the past [17], [18]. On the other hand, because of the process of virus-driven evolution of host innate immunity, it is possible to estimate the true evolutionary age of viruses by tracking and dating the evolution of antiviral genes [19]. Ideal candidate genes for this type of analysis are restriction factors, cellular proteins that coordinate the cell-intrinsic innate immune response to virus infections. Moreover, if the virus encodes an antagonist of the restriction factor, and the interactive interface between host and viral factors is known, then evolution at the site(s) of interaction can be used to infer past instances of infection [20].

The host restriction factor APOBEC3G (A3G) is a cytidine deaminase that restricts lentivirus replication by hypermutating viral DNA and by inhibiting reverse transcription [21]–[23]. To overcome this block, all known primate lentiviruses encode the accessory protein Vif [5], which links A3G to a cellular E3 ubiquitin ligase complex and accelerates its turnover at the proteasome [24]–[27]. The early birth and ongoing retention of *vif* within all circulating primate lentiviruses [7], [28] suggest that antagonism of A3G is crucial to lentivirus spread and survival. Therefore, *A3G* is a likely substrate for signatures of lentivirus-driven selection, from which a detailed account of past viral challenges can be reconstructed.

Previously, we studied the co-evolution of *A3G* and *vif* in the setting of natural SIVagm infections in African green monkeys (AGM). We found that the *A3G* is subject to recent diversifying selection in wild monkey populations, with single nucleotide polymorphisms (SNPs) encoding charge altering amino acid changes at surfaces targeted by Vif [29]. Our data support that these naturally occurring mutations in *A3G* were selected to allow evasion of SIVagm Vif proteins, implicating Vif as the selective pressure responsible. Adaptive evolution at the A3G-Vif interface in recently diverged primate populations implies that some modern SIV infections can incur a cost to host fitness, whether it be overt immunodeficiency or more subtle phenotypes that decrease host survivability or fertility [29].

In the present study, we trace the co-evolution of A3G and Vif through deep evolutionary time using an array of diverse primate species and SIV isolates. Our work allow us to provide a minimum age estimate for simian primate lentivirus infections, as well as an illustration of the dynamic flux of a host-pathogen interaction over time. We find that multiple species of the Old World Monkey (OWM) subfamily *Cercopithecinae* possess mutations in the Vif interaction site of *A3G* and that each allows escape from antagonism by Vif proteins. The recurrence and deep ancestry of such mutations suggest that a lentivirus encoding Vif existed at least 5–6 MYA. In response, contemporary Vif proteins have counter-evolved to these various Vif-resistant forms of A3G by tolerating amino acid variation at the canonical Vif interaction site. Moreover, we reveal an even older ancestral insertion event in the N-terminus of *A3G* of the *Colobinae* subfamily that conceals the Vif-binding site and precludes interaction with Vif proteins, suggesting that lentiviruses may have infected primates as much as 12 MYA. Coincident with this unique host adaptation, a Vif protein from a lentivirus currently infecting one of the *Colobinae* species has evolved to recognize a novel surface of A3G. Furthermore, we highlight the adaptability of lentiviral Vif proteins and the possible impact that this evolution may have on cross-species transmission and virus emergence. For example, Vif from a lentivirus infecting sooty mangabeys (SIVsm) and its descendants (SIVmac and HIV-2), exhibit exceptional breadth, possibly explaining in part how SIVsm was able to successfully colonize both humans and macaque species. Together, these data suggest that infections of OWM by primate lentiviruses are older than previous thought, driving selective changes in antiviral genes of their natural hosts and inciting an evolutionary arms race that continues to this day.

## Results

### The Vif binding site of A3G is adaptively diversifying in the Cercopithecinae subfamily of Old World Monkeys

**Figure 1:**
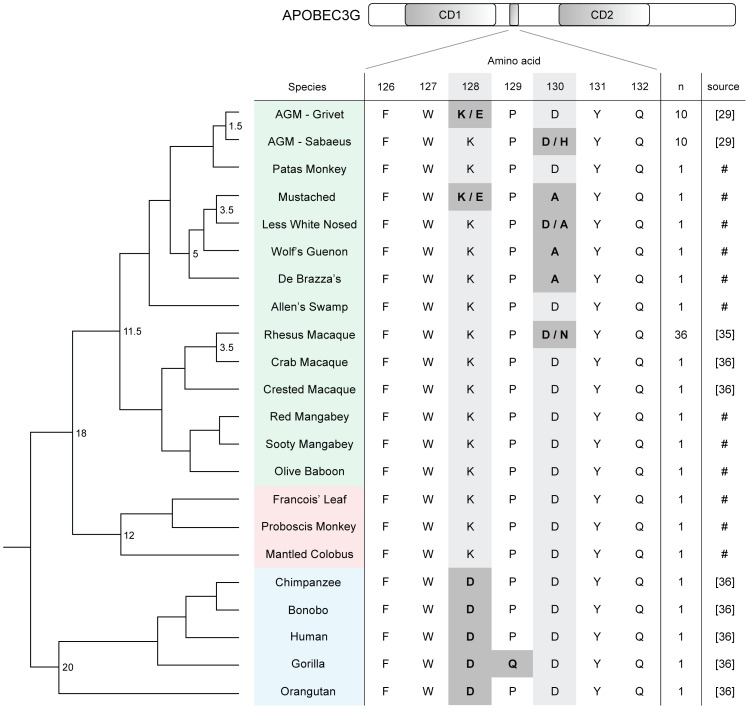
Diversifying selection of the Vif binding site of *APOBEC3G* in the *Cercopithecinae* subfamily of Old World Monkeys. A partial primate species phylogeny [37] is depicted as a cladogram and accompanied by partial amino acid sequences of respective A3G orthologs. Approximate divergence times (in millions of years) are placed at relevant ancestral nodes. Select residues comprising the putative site targeted by Vif proteins are shown. Primates of the order *Catarrhini*, consisting of hominoids (blue) and Old World Monkeys, are included. Old World Monkeys are subdivided into the *Cercopithecinae* (green) and *Colobinae* (red) subfamilies. Sources of sequences previously reported elsewhere are indicated by reference number. # = new sequences reported in this study, n = number of individuals analyzed per species.

The bulk of known SIVs that circulate in the wild have been found within the *Cercopithecinae* subfamily of OWM, a group that includes AGM, mangabeys, macaques, and members of the *Cercopithecus* genus (collectively known as guenons) [5]. We previously identified naturally occurring amino acid changes within ^126^FWKPDYQ^132^ of A3G in AGM populations, a motif that is critical to the interaction between A3G and Vif [30]–[34]. Specifically, polymorphisms at codons 128 and 130 of AGM A3G were found to be adaptive because they confer resistance to Vif-mediated antagonism [29]. In order to further characterize the age and distribution of the genetic conflict between *A3G* and *vif*, we asked whether adaptive signatures in *A3G* were common to a wide range of OWM hosts. Full-length *A3G* was amplified from single representatives of OWM species, including members of the divergent *Colobinae* subfamily. In addition, the dataset was supplemented by previously published sequences from NCBI GenBank [35], [36] (Figure 1).

We found that mutations at codons 128 and 130 of *A3G* have emerged independently in several primate lineages (Figure 1). In particular, a D130A mutation was detected in four members of the *Cercopithecus* genus: the De Brazza's monkey (*C. neglectus*), Wolf's guenon (*C. wolfi*), the lesser white nosed monkey (*C. petaurista*), and the mustached guenon (*C. cephus*) (Figure 1). We found D130A homozygosity in three of the four species, while the lesser white nosed monkey is heterozygous. The presence of the D130A mutation in four separate guenon species suggests that it has approached fixation since emerging in the ancestor of the *Cercopithecus* genus 5–6 MYA [37]. In addition to D130A, one allele of A3G from the mustached guenon contains a K128E mutation, a Vif-blocking SNP previously observed in a proportion of grivet monkeys (AGM) (Figure 1) [29].

Previous work has demonstrated that *A3G* is undergoing adaptive evolution in primates, as measured by the relative rates of non-synonymous variation (dN) and synonymous variation (dS) [36], [38]. However, given the additional interspecies and intraspecies *A3G* sequences reported in this study (Figure S1 and S2), we reexamined the gene's evolutionary history within the OWM clade to determine if the pattern of non-synonymous mutation is suggestive of selection, and moreover, of exposure to a common selective pressure [39], [40]. In agreement with previous efforts, our results indicate that the *A3G* locus as a whole is evolving according to diversifying (positive) selection in OWM (Table S1). A model that allows sites to evolve under selection (M8) provides a significantly better fit to the molecular data than does a model of neutral evolution (M7) (Table S1). Using the mixed-effected model of evolution (MEME) [41] to identify specific residues that are subject to diversifying selection, we find strong signals of selection originating from 13 codons spread throughout the length of the gene, including codons 128 and 130 of the Vif binding site (p = 0.0496 and p = 0.0493, respectively) (Table S2). The random effects likelihood (REL) analysis also identified codons 128 and 130 as being under positive selection, with both sites displaying dN/dS>1 with high posterior probabilities (>0.99) (Table S2). Overall, our analyses support that the constellation of mutations identified at codons 128 and 130 of *A3G* result from natural selection, suggesting that a genetic conflict between *A3G* and *vif* is proceeding in multiple species of OWM.

### Ancient, high frequency SNPs in OWM A3G allow escape from Vif-mediated degradation

To test whether the D130A and K128E mutations in A3G are adaptive by affecting susceptibility to Vif-mediated antagonism, we measured the sensitivity of A3G variants to a panel of Vif proteins, including autologous isolates (derived from viruses naturally circulating in a given host species) and heterologous isolates (those derived from other host species). Since viral adaptation can mask the adaptive phenotype of genetic mutations of the host, the use of a broad spectrum of Vif proteins, each with a different adaptive history, allows us to assess how mutations in A3G affect sensitivity to antagonism.

**Figure 2:**
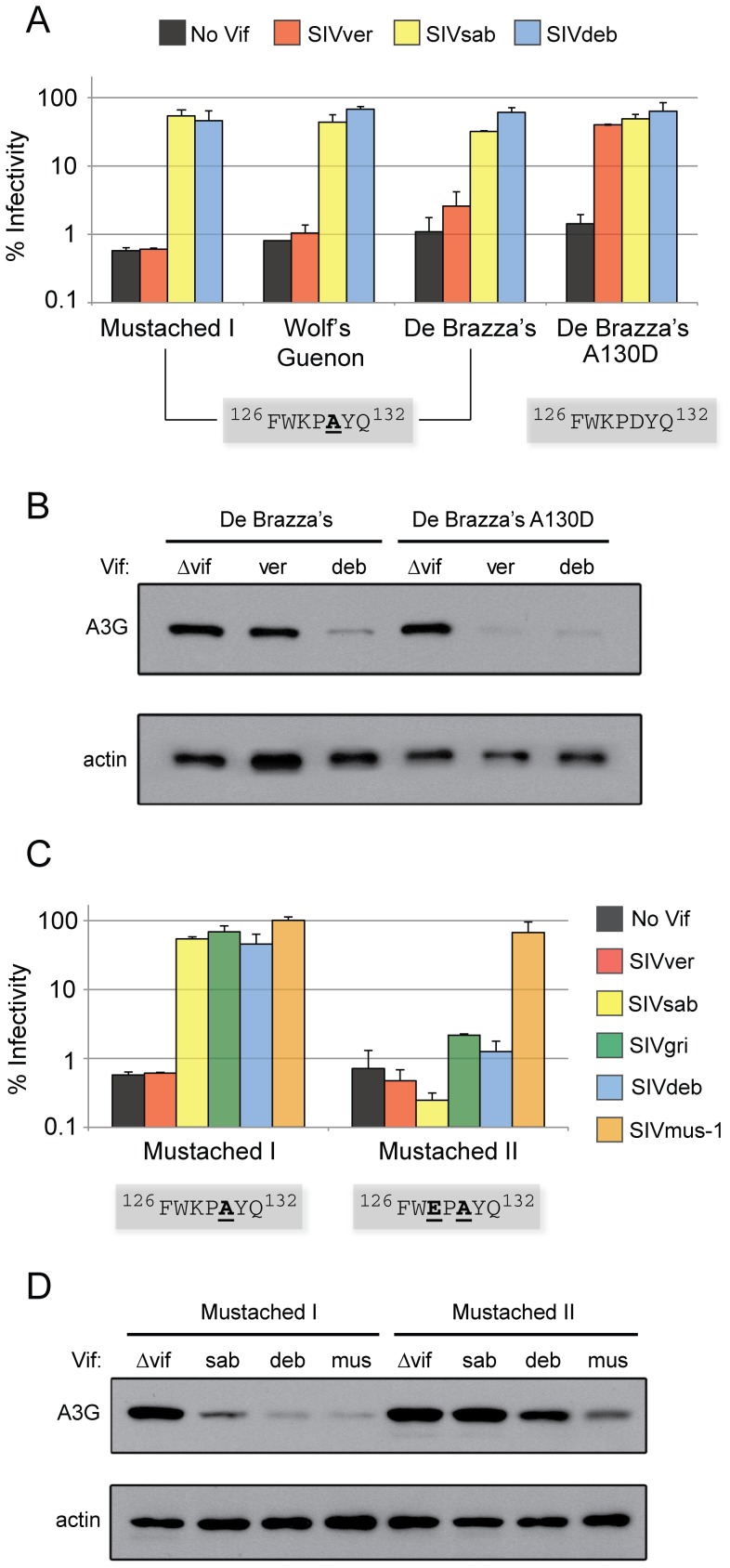
Adaptive evolution of *A3G* at codons 128 and 130 allows escape from Vif-mediated antagonism. Single-round infectivity assays were performed with HIV-1ΔVif and HIV-1 expressing SIV Vif proteins produced in the presence of A3G variants from monkeys of the *Cercopithecus* genus in (A) and (C). Infectivity of viruses is reported as a percentage, relative to infectivity in the absence of A3G (100%). Error bars indicate standard deviation from the mean of two independent transfection experiments (six infection replicates in total). The Vif binding site of each A3G variant is depicted in grey boxes. (A) and (C) The primate *A3G* gene used is listed under the graph, while the source of the *Vif* genes were either no Vif (black box), SIVagm.ver (orange), SIVagm.sab (yellow), SIVdeb (blue), SIVagm.gri (green), or SIVmus-1 (golden). Anti-HA western blot analysis was used to measure A3G expression in virus producer cells in (B) and (D). Anti-ß-actin served as protein loading controls.

The antiviral activity of A3G from De Brazza's monkey, Wolf's guenon, and mustached guenon was measured by co-expressing each variant with Vif-deficient HIV-1. All A3G variants inhibit infectivity of the virus by more than 100-fold relative to virus produced in the absence of A3G, demonstrating that the antiviral activity of A3G orthologs has been conserved despite the observed variation (Figure 2a).

Using recombinant HIV-1 virus engineered to express SIV *vif* genes, we found that De Brazza's A3G is resistant to antagonism by Vif from SIVagm.Ver, a heterologous isolate that infects vervet monkeys (AGM) (Figure 2a). However, reversion of D130A (A130D, restoration of ancestral ^128^KPD^130^) renders De Brazza's A3G fully sensitive, demonstrating that a single alanine residue at position 130 allows escape from antagonism. Conversely, Vif from the virus that naturally circulates among De Brazza's monkeys in the wild, SIVdeb [42], readily antagonizes De Brazza's A3G despite the D130A adaptation. A3G from two other members of the *Cercopithecus* genus (Mustached guenon (allele I) and Wolf's guenon) exhibits the same sensitivity as De Brazza's A3G (Figure 2a). These data suggest that the emergence of D130A in the ancestor of the *Cercopithecus* genus drove Vif (e.g. SIVdeb Vif) to adapt to this highly prevalent A3G variant. Furthermore, De Brazza's A3G is also sensitive to SIVagm.Sab Vif, which antagonizes A3G carrying D130A or A130D. This activity is likely the result of prior adaptation to the D130H polymorphism in A3G from sabaeus monkeys (AGM), as previously described [29]. Western blot analysis confirms that antagonism of A3G variants by Vif results in depletion of intracellular A3G protein. De Brazza's A3G expression is reduced substantially in the presence of SIVdeb Vif, relative to expression in the absence of Vif, whereas SIVagm.Ver Vif has no impact (Figure 2b). Upon reversion of the D130A mutation, however, SIVagm.Ver Vif is capable of depleting A3G levels (Figure 2b). These results suggest that the derived D130A mutation was selected 5–6 MYA to evade ancestral SIV Vif proteins.

### Recent virus-driven evolution at residue 128 of A3G

One variant of A3G specific to the mustached guenon, the ^128^EPA^130^ variant encoding K128E in addition to D130A (Mustached II), is resistant to four heterologous Vif proteins: SIVagm.Ver Vif, SIVagm.Sab Vif, SIVagm.Gri Vif, and SIVdeb Vif isolates (Figure 2c). This demonstrates that K128E, like D130A, prevents Vif-mediated antagonism. However, this variant of A3G is sensitive to Vif from SIVmus-1, indicating that at least one of the three lentivirus strains currently circulating in mustached guenons [43], [44] has counter-evolved while adapting to this species (Figure 2c). Concordantly, only SIVmus-1 Vif depletes expression levels of the ^128^EPA^130^ variant (Mustached II), while the ^128^KPA^130^ variant (Mustached I) common to other *Cercopithecus* monkeys is degraded by several Vif proteins (Figure 2d). These data demonstrate that K128E and D130A were selected at different times during primate evolution to prevent Vif-mediated antagonism of A3G, with the latter occurring 5–6 MYA in the common ancestor of the *Cercopithecus* genus and the former appearing recently in a single species (the mustached guenon). Recurrent virus-driven evolution of *A3G* over time suggests that natural host species are engaged in a prolonged, antagonistic relationship with lentiviruses.

### Broad specificity of some Vif proteins may facilitate cross-species transmission

**Figure 3:**
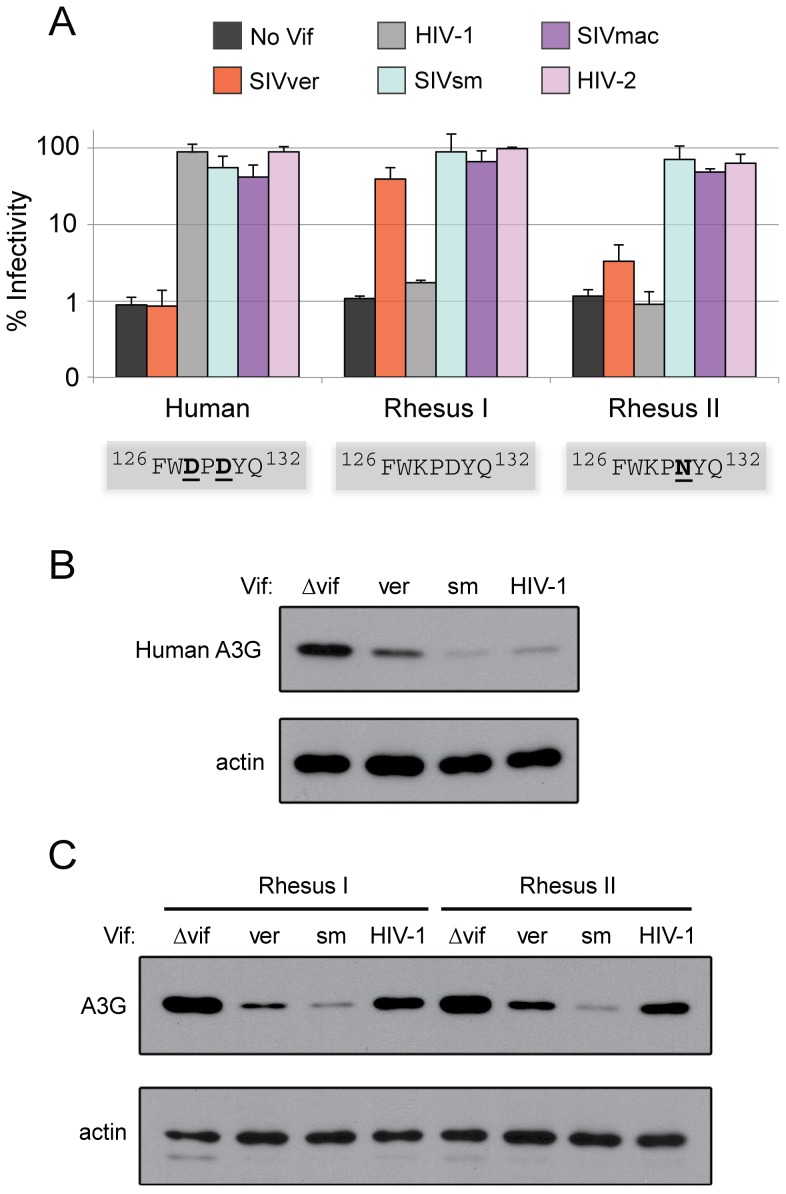
Broad and potent activity of Vif from SIVsm and its descendants allow antagonism of rhesus and human A3G variants. Single-round infectivity assays were performed with HIV-1ΔVif and HIV-1 expressing SIV Vif proteins produced in the presence of A3G variants from human and rhesus macaques (A). Infectivity of viruses is reported as a percentage, relative to infectivity in the absence of A3G (100%). Error bars indicate standard deviation from the mean of two independent transfection experiments (six infection replicates in total). The Vif binding site of each A3G variant is depicted in grey boxes. The primate *A3G* gene used is listed under the graph, while the source of the *Vif* genes were either no Vif (black box), SIVagm.ver (orange), HIV-1 (grey), SIVsm (light blue), SIVmac (dark purple), or HIV-2 (light purple). Anti-HA western blot analysis was used to measure human A3G expression in (B) and rhesus A3G expression in (C). Anti-ß-actin served as protein loading controls.

While most examples of variation at the Vif binding site of *A3G* were identified in natural hosts of modern SIV strains, we also found unique variation among rhesus macaques (*Macaca mulatta*). Captive macaques have experienced simian AIDS stemming from accidental and experimental cross-species transmissions of SIVsm (giving rise to SIVmac) in the 1970s [45], [46], but they are not thought to harbor a lentivirus in wild Asian habitats. Using a previously published dataset from 36 Indian-origin rhesus macaques, a D130N polymorphism in *A3G* was identified in 59/74 (80%) of chromosomes examined [35] (Figure 1). Like the D130H and D130A mutations observed in sabaeus monkeys and members of the *Cercopithecus* genus, respectively, rhesus A3G encoding D130N (variant ^128^KPN^130^) resists antagonism by SIVagm.Ver Vif (Figure 3a right side). Since SIVsm was able to cross-transmit into both humans and macaques, with both species exhibiting specific variation at the Vif-binding site of A3G (Figure 1), we tested the activity of SIVsm Vif. Similarly to the macaque-adapted strain SIVmac, Vif from SIVsm is capable of antagonizing rhesus A3G despite the D130N mutation (Figure 3a). Western blot analysis demonstrates that both variants of rhesus A3G are depleted by SIVsm Vif, but not by HIV-1 Vif, while SIVagm.Ver Vif is only capable of degrading the variant encoding the ancestral ^128^KPD^130^ motif (Rhesus I) (Figure 3c). Furthermore, human A3G is susceptible to antagonism by HIV-2 Vif as well as Vif from SIVsm, in agreement with a prior report [47], but not SIVagm.Ver Vif (Figure 3a left side and 3b). These data demonstrate that SIVsm Vif, in exhibiting broad cross reactivity for the A3G substrate, was ‘pre-optimized’ to target both rhesus A3G and human A3G prior to cross-species transmission. Moreover, this capacity for widespread antagonism has been maintained by SIVmac and HIV-2 following emergence in rhesus macaques and humans, respectively.

Our characterization of SIV Vif proteins suggests that some have evolved to tolerate variation at the Vif-binding site of A3G. To determine *vif* counter-evolution produces antagonists that continue to rely on residues 128 and 130 or whether it shifts the stage of the genetic conflict to distinct surfaces on A3G, we tested the activity of Vif proteins against seven A3G variants representing each variation of the Vif-binding site. While the range of A3G variants targeted by each Vif varies, no Vif was capable of antagonizing the full spectrum (Table 1). SIVmus-1 Vif fails to inhibit human A3G and only minimally inhibits the two AGM A3G variants. Furthermore, Vif from SIVagm.Sab recognizes A3G from AGM, rhesus, and De Brazza's, but cannot tolerate ^128^EPA^130^ present in mustached guenon A3G. The broadest acting Vif species, encoded by SIVsm and its descendants (SIVmac and HIV-2), exhibit specificity for nearly all variants of A3G reported here. However, all three are defective at targeting the ^128^KPH^130^ variant found in sabaeus monkeys (Table 1). These data indicate that, despite differences in substrate specificity, Vif isolates from viruses infecting *Cercopithecinae* monkeys share a dependency on residues 128 and 130 for antagonism of A3G. Therefore, Vif is most likely the selective agent responsible for the recurrent selection of ‘escape’ mutations at these positions (Figure 1).

**Table 1:** Sensitivity of A3G variants to a spectrum of diverse SIV Vif proteins.

	SIVver	SIVsab	SIVmus	SIVsm	SIVmac	HIV-2	SIVolc
Rhesus I ^128^KPD^130^	39+/−16	51+/−6	60+/−5	89+/−59	63+/−24	98+/−5	<1
Rhesus II ^128^KPN^130^	3+/−2	66	59	70+/−34	49+/−4	63+/−19	<1
De Brazza's ^128^KPA^130^	2+/−1	32+/−1	79+/−39	50	90	89+/−29	1
Mustached II ^128^EPA^130^	<1	<1	68+/−28	105	52	70	<1
AGM Sabaeus ^128^KPH^130^	<1	64+/−22	8	<1	17	3+/−1	<1
AGM Grivet ^128^EPD^130^	<1	<1	5	32	55	83+/−26	<1
Human ^128^DPD^130^	<1	<1	2	55+/−23	42+/−18	89+/−14	<1
Colobus ^128^KPD^130^	1	3+/−1	14+/−2	2	1	3+/−2	75+/−30

Infectivity of viruses produced in the presence of various A3G variants is reported as a percentage, relative to infectivity in the absence of A3G (100%). Standard deviations from the mean of two to three independent transfection experiments (six to nine infection replicates in total) are reported when available. Rows are headed with OWM A3G variants with the amino acid sequence between residues 128–130 indicated. Columns are headed with SIV Vif isolates (the name of the virus from which the *Vif* gene is taken is listed).

### A multi-residue insertion that blocks Vif emerged approximately 12 MYA in A3G of the Colobinae ancestor

In studying the species-specificity of SIV Vif proteins, we found that A3G from the mantled colobus monkey (*Colobus guereza*) is widely resistant to most SIV Vif proteins (Table 1), despite carrying the ancestral ^128^KPD^130^ at the Vif binding site (Figure 1). This observation suggests that residues in A3G lying outside of the canonical Vif binding motif can govern susceptibility to antagonism. The mantled colobus species (hereafter referred to as colobus) belongs to the *Colobinae* subfamily of OWM, a group of primates that diverged from *Cercopithecinae* about 18 MYA [37]. It is naturally associated with a specific SIV strain termed SIVcol, and this is the case for other closely related species (SIVwrc in western red colobus and SIVolc in olive colobus) [48]–[52]. In testing the sensitivity of colobus A3G to Vif from viruses naturally associated with *Colobinae* hosts, we found that SIVolc Vif was unique in its ability to target it for destruction (Figure S3). In fact, SIVolc Vif antagonizes solely colobus A3G and not A3G from any other primate species tested (Table 1). Conversely, SIVagm.Sab Vif exhibits the opposite specificity, readily counteracting AGM A3G and several OWM A3G orthologs but not colobus A3G (Table 1).

**Figure 4:**
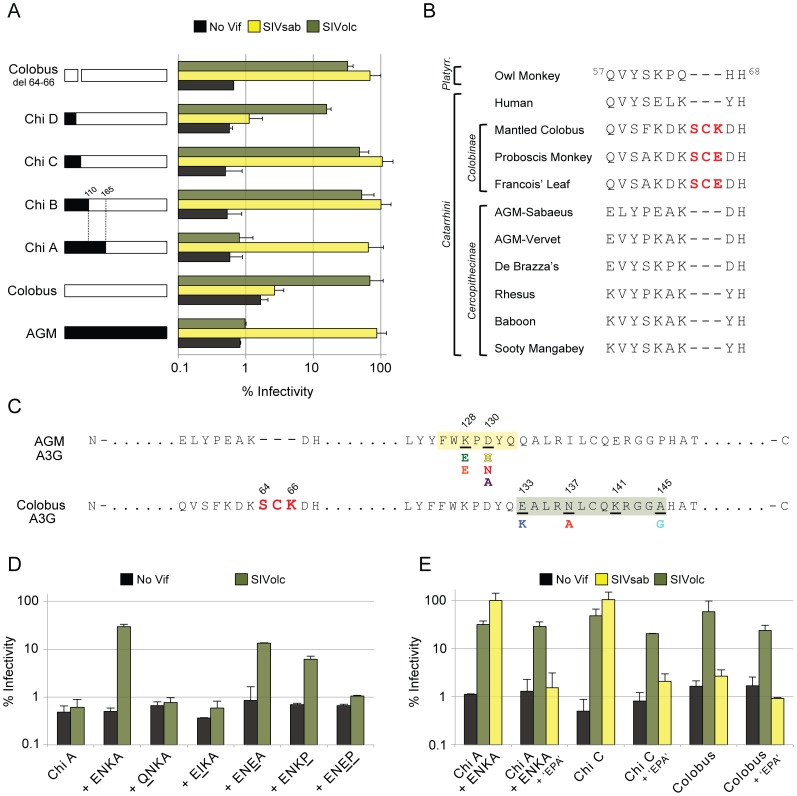
A multi-residue insertion that blocks Vif emerged 12 MYA in *A3G* of the *Colobinae* ancestor. (A) Single-round infectivity assays were performed with HIV-1ΔVif and HIV-1 expressing SIV Vif proteins produced in the presence of colobus/AGM chimeric A3G proteins. Infectivity of viruses is reported as a percentage, relative to infectivity in the absence of A3G (100%). Error bars indicate standard deviation from the mean of two independent transfection experiments (six infection replicates in total). (B) Partial protein alignment of A3G orthologs reveals a three-residue insertion (red) unique to members of the *Colobinae* subfamily of OWM. Members of primate parvorders *Catarrhini* (Old World Monkeys and Hominoids) and *Platyrrhini* (New World Monkeys) are included. Old World Monkeys are further divided into subfamilies *Cercopithecinae* and *Colobinae*. (C) Residues of A3G responsible for differential sensitivity to SIVsab and SIVolc Vif. The canonical Vif binding site is boxed in yellow. Divergent character states independently selected at residues 128 and 130 (underlined) of *Cercopithecinae A3G* are displayed below. The three-residue insertion in the N-terminus of colobus A3G is bolded in red. The residues of A3G required for antagonism by SIVolc Vif, as found in this study, are underlined and boxed in green. Divergent character states at residues 133, 137 and 145 identified in *Colobinae A3G* are displayed below. (D) and (E) Single-round infectivity assays were performed with HIV-1ΔVif and HIV-1 expressing SIVolc Vif produced in the presence of mutated colobus/AGM chimeras.

To learn how colobus A3G remains resistant to nearly all Vif proteins except SIVolc Vif, we constructed chimeric A3G proteins containing portions of the N-terminus of AGM A3G and the C-terminus of colobus A3G. These chimeras were co-expressed with virus encoding SIVagm.Sab Vif or SIVolc Vif to test for sensitivity to antagonism. The critical constructs are shown in Figure 4a. Chimera C and chimera D differ by only seven amino acids, yet the former is sensitive to SIVagm.Sab Vif while the latter is resistant (Figure 4a). Interestingly, a multi-residue insertion unique to members of the *Colobinae* subfamily is contained within this sequence (Figure 4b). Upon removal of the insertion (^66^SCK^68^) from wild-type colobus A3G, a full gain in sensitivity to SIVagm.Sab Vif is achieved (Figure 4a, compare Colobus del 64–66 to Colobus A3G). Therefore, a three amino acid insertion that emerged in the N-terminus of A3G in the *Colobinae* ancestor prevents antagonism by Vif. The Vif-blocking activity of ^66^SCK^68^ is context dependent in that it only blocks other Vif proteins when in combination with residues 66–199 of colobus A3G (data not shown).

In comparison to SIVagm.Sab Vif, SIVolc Vif displayed different specificities for the same chimeric A3G proteins, demonstrating that it has diverged to target distinct surfaces of the A3G substrate (Figure 4a, compare Chi B to Chi A). Mutagenesis of residues within 110–165 that are divergent between AGM A3G and colobus A3G reveal that E133, N137, K141, and A145 (depicted as ‘ENKA’) are major recognition determinants of SIVolc Vif (Figure 4c and 4d). Single mutations of E133 or N137 alone completely prevent antagonism by SIVolc Vif, while mutation of K141 and A145 in combination also blocks antagonism (Figure 4d). Interestingly, residues 133, 137, and 145 are divergent between members of the *Colobinae* subfamily, suggesting that this motif may be diversifying in response to Vif from SIV infecting these primates (Figure 4c and Figure S2).

In order to determine if SIVolc Vif antagonizes A3G independently of the “canonical” Vif interaction motif involving residues 128 and 130, we tested its ability to antagonize A3G encoding against naturally occurring mutations at these sites. Indeed, we found that A3G constructs that are sensitive to SIVolc Vif remain so after the introduction of the K128E and D130A mutations (the ^128^EPA^130^ motif found in mustached A3G) (Figure 4e). Conversely, these mutations completely abrogated antagonism by SIVagm.Sab Vif (Figure 4e). Therefore, SIVolc Vif has diverged to utilize unique surfaces of A3G while adapting to its natural host, targeting residues that are divorced from those targeted by all other Vif proteins studied to date.

## Discussion

We have used the approach of studying virus-driven host evolution to discern the minimum age for the association between OWM and SIV at 5–6 MYA, and possibly 12 MYA. Moreover, using a broad range of primates and SIV isolates, we have identified both recurrent and novel interactions between Vif with A3G. Finally, our data suggests that the broad specificity exhibited by some Vif proteins may facilitate cross-species virus transmission events.

### Adaptive evolution at the Vif binding site of A3G in Cercopithecinae monkeys is a molecular beacon for past lentivirus infections

Using maximum likelihood methods, we reveal that the region of *A3G* targeted by Vif has been independently diversifying in several primate lineages. While a previous report of positive selection in primate *A3G* concluded that Vif (and by extension, lentiviruses) did not play a major role in the gene's evolution [36], the data set used was limited in the number of natural hosts of SIV. Using a data set enriched for OWM species, we discover recurrent charge-altering mutations at residues 128 and 130 of the Vif interaction site that are evolving under positive selection. Importantly, *in vitro* infections reveal that single amino acid changes affect sensitivity to Vif-mediated degradation. Thus, in a remarkable case of convergent evolution, diverse Vif isolates have independently selected for mutations at the same amino acid residues of A3G in multiple lineages of simian primates. While this scenario is often loosely inferred from genetic data alone, our functional demonstrations that convergent amino acid changes affect the host-virus interface between Vif and A3G provide strong support that lentiviruses can shape the evolution of the hosts that they infect. Importantly, virus-driven evolution of *A3G* is apparent in ancestral primate species that existed many millions of years ago as well as extant primate species, suggesting that lentiviruses are an enduring selective pressure.

It is important to note that we cannot exclude the possibility that this region of *A3G* is subject to selective pressures other than lentiviral Vif. However, recurrent selection at precisely the same sites targeted by most SIV Vif proteins supports that the selective agent responsible is a Vif-encoding element. These data suggest that Vif drives the emergence of ‘escape’ mutations in *A3G* that allow evasion of Vif-mediated degradation, which in turn promotes *vif* counter-evolution and the perpetuation of a genetic conflict between host and virus.

What remains unresolved is whether or not the ancient pathogens inferred by this study are the direct ancestors of contemporary SIV strains. We raise two possibilities: 1) extant lentiviruses are themselves ancient, having coexisted continuously with their specific hosts for millions of years, or 2) extant lentivirus infections are young, such that adaptive evolution at the A3G-Vif interface was driven by lentiviruses that no longer exist (paleoviruses). The second scenario posits that modern SIV strains may not necessarily bear semblance to the lentiviruses that drove selection in *A3G*, a distinct possibility given the prevalence of cross species transmission [53], dual lentivirus infections and circulating recombinant forms [43], [54], [55], and virus lineage extinction [56]. That is, the evolutionary histories of natural host species may be punctuated by periodic lentivirus infections rather than by a single, enduring lentiviral threat.

Along with our previous discovery of adaptive evolution in *A3G* of AGM [29], the emergence of ‘escape’ mutations in members of the *Cercopithecus* genus yield insight into the age and pathogenic potential of SIV infections in natural host species. The D130A mutation common to the four members of the genus indicates that *Cercopithecus* ancestors were exposed to a form of SIV prior to speciation, one that impacted host fitness and selected for adaptive mutations in innate immunity. Furthermore, the subsequent emergence of E128K within one of those species, the mustached guenon, suggests the selective pressure applied by SIV is not only ancient but also *ongoing*. This particular mutation is unlikely to be found in other members of *Cercopithecus*, since SIVmus-1 is unique in its ability to degrade it. Vif from SIVdeb, a strain infecting a closely related host, the De Brazza's monkey, does not tolerate variation at this site (Figure 2c). Therefore, E128K likely represents a more recent adaptive change than the D130A mutation common to the genus. The sequential emergence of two mutations that each allowed escape from Vif proteins suggests that the mustached guenon lineage has been subjected to continuous (scenario 1, above) or periodic (scenario 2) selective pressure by SIV since diverging from other members of the genus 5–6 MYA. This is considerably older than previous phylogenetic analyses have indicated, and presents an alternative route to dating viral infections that does not suffer from the limitations of virus sequence-based methods [17], [18]. Moreover, our analysis of virus-driven evolution provides an age estimate of SIV in the ancestors of present day natural hosts that complements the finding of an endogenous lentivirus in lemurs [14], [15], which also suggested an ancient association between primates and lentiviruses extending back at least 4 million years.

Another example of variation in the Vif-binding motif of A3G was found in the rhesus macaque, a species of Asian descent that is not known to carry a circulating lentivirus. The genetic heterogeneity of rhesus macaques is also evident in the *TRIM5* gene, which is highly polymorphic and gives rise to seven distinct variants with anti-lentivirus activity [57]. Moreover, one allele encodes a TRIM5-CypA fusion protein that restricts HIV-2 and SIVagm, suggesting that it was selected for 5–6 MYA by a virus with similar characteristics [58]–[61]. Together with the data presented here on *A3G* polymorphism, the rhesus genome abounds with genetic clues alluding to a former lentiviral presence that is now extinct. However, further study of diverse Asian primates is needed to support this speculation.

### Broad specificity of SIV Vif originating from Cercopithecinae hosts with polymorphic A3G

Our results indicate that, upon adaptation to one or more variants of A3G in a polymorphic host species, SIV evolves Vif proteins with broader specificity. That is, by counter-evolving to antagonize resistant variants of A3G from their respective host species, SIV Vif gains the ability to target A3G variants present in other species. This is evidenced by SIVagm.Sab Vif which, having adapted to target ^128^KPD^130^ and ^128^KPH^130^ variants of A3G in sabaeus monkeys, exhibits the capacity to antagonize the ^128^KPA^130^ variant in *Cercopithecus* monkeys and the ^128^KPN^130^ variant in Rhesus macaques (Figure 2a, Table 1). This is in stark contrast to the related SIVagm.Ver Vif, which solely antagonizes A3G bearing the ^128^KPD^130^ motif ancestral to OWM. Furthermore, SIVmus-1 Vif has adapted to persist among mustached guenons, which have presented adaptive variation at both residues 128 and 130 of A3G. In doing so, SIVmus-1 Vif can cross-react with a broad array of A3G orthologs that present different combinations of characters at these two sites (Table 1). Likewise, in the cases of SIVmac and HIV-2, the broad activity of their Vif proteins may have been pre-determined by events that played out in sooty mangabey populations (Table 1). Our data suggest that SIVsm was ‘pre-optimized’ to target both rhesus A3G and human A3G prior to cross-species transmission, and that this activity has been maintained by SIVmac and HIV-2 following emergence in rhesus macaques and humans, respectively. Although we did not see “escape” mutations in the sooty mangabey *A3G*, we did not have a large enough population sample to detect polymorphisms that were not fixed or at high frequency. Nonetheless, we believe that the cross-reactivity or ‘promiscuity’ of Vif proteins, as exemplified by SIVsm Vif, can shine light on the unique adaptive history of lentivirus strains and provide clues about the *A3G* diversity of its host species.

### A novel insertion in Colobus A3G has altered Vif targeting preferences

While the canonical Vif binding site of A3G was conserved in members of the divergent *Colobinae* subfamily of OWM, we found that a multi-residue insertion (^66^SCK/E^68^) in the N-terminus renders A3G resistant to nearly all Vif proteins. As of yet there is no crystal structure of the A3G-Vif interaction, but our experimental results demonstrate how the insertion disrupts the ability for Vif to counteract A3G. Upon removal of these three residues from colobus A3G, this variant becomes sensitive to antagonism by SIVagm.Sab Vif, revealing that all the determinants necessary for binding and degradation are intact elsewhere in the protein. Thus, we hypothesize that the ^66^SCK^68^ insertion serves to conceal the typical Vif binding site by altering protein conformation and masking distal epitopes necessary for Vif-mediated antagonism. Furthermore, our studies using chimeric A3G proteins demonstrate that the insertion functions as such only in the context of colobus A3G. Thus, the multi-residue insertion may represent an alternative strategy to evade antagonism by Vif proteins that evolved within the *Colobinae* lineage. Our functional analysis suggests that this adaptive feat drove the evolution of SIV Vif proteins with different targeting preferences. That is, in adapting to the presence of the ^66^SCK^68^ insertion in colobus A3G, SIVolc Vif has evolved to recognize a patch of residues offset from the binding site preferred by other Vif proteins. Therefore, the stage of the genetic conflict between A3G and Vif has shifted at least once during primate evolution. The consequences of this switch in substrate recognition may already be evolutionarily apparent, as residues of A3G important for SIVolc Vif-mediated antagonism are divergent in *Colobinae* species used in this study (Figure 4c).

In summary, the data reported herein allow us to infer the presence of lentiviruses that applied pathogenic selective pressure at different points in primate evolutionary history. In a marked display of convergent evolution, two residues of A3G that coordinate an interaction with SIV Vif are diversifying in multiple primate species. Moreover, a divergent strategy of Vif-evasion has emerged in a separate branch of the primate phylogeny, giving rise to a second genetic conflict and altering the interface of the A3G-Vif interaction. The pattern of adaptive mutation suggests that SIV has been infecting OWM on timescale of millions of years.

## Materials and Methods

### PCR amplification of primate A3G and generation of A3G expression plasmids

The following fibroblast or lymphoid cell lines derived from primate species were obtained from Coriell Cell Repositories (Camden, NJ): patas monkey (*Erythrocebus patas*; AG06116A), mustached guenon (*Cercopithecus cephus*; PR00527), lesser white nosed monkey (*Cercopithecus petaurista*; PR00949), Wolf's guenon (*Cercopithecus wolfi*; PR01241), De Brazza's monkey (*Cercopithecus neglectus*; PR01144), Allen's swamp monkey (*Allenopithecus nigroviridis*; PR01231), red capped mangabey (*Cercocebus torquatus*; PR00485), sooty mangabey (*Cercocebus atys*; G077), Francois' leaf monkey (*Trachypithecus francoisi*; PR01099), proboscis monkey (*Nasalis larvatus*; PR00674), and the mantled colobus monkey (*Colobus guereza*; PR00980). The following transformed lymphoid cell lines were obtained from the NIH Nonhuman Primate Reagent Resource: Olive baboon (*Pabio anubis*, GAG-LCL). Whole RNA was extracted using the RNeasy Mini Kit (QIAGEN). Full-length *A3G* was amplified via one-step RT-PCR with the SuperScript III Reverse-Transcriptase Kit (Invitrogen) using primers specific for OWM A3G (FOR 5′-ATG AAG CCT CAA ATC AGA AAC ATG G-3′, REV 5′-CAG TTT CCC TGA TTC TGG-3′). Bulk PCR product was subcloned, and six to ten clones were sequenced. If two distinct *A3G* sequences were detected, the individual was considered to be heterozygous. OWM *A3G* sequences were appended with a 5′ hemagglutinin (HA) tag by PCR and cloned into the mammalian expression vector pcDNA3.1.

### Evolutionary analysis of OWM A3G

Alignment of newly derived *A3G* nucleotide sequences from OWM plus those previously published in NCBI GenBank was executed in ClustalW (Figure S3). Phylogenetic reconstruction by maximum likelihood was performed with the web-based version of PhyML [62] (Figure S2). The resulting *A3G* phylogeny and the currently accepted phylogeny of OWM species [37] are similar but not identical, most likely due to rampant selection. The *A3G* phylogeny does not recapitulate that macaques, baboons, and mangabeys share a single common ancestor, and some intraspecies variants do not share immediate common ancestry (De Brazza's and red capped mangabey). Moreover, Allen's swamp monkey is placed ancestral to the Cercopithicini tribe (AGM, Patas, guenons), reflecting a previous classification [63]. A phylogeny consistent with the currently accepted phylogeny of OWM species (the placement of Allen's swamp monkey is the exception) (Figure S2a) was uploaded to the Codeml program (of the PAML suite) and to the web-based version of HyPhy (DataMonkey, www.datamonkey.org) for molecular evolution analysis. Given an alignment and phylogenetic tree of primate *A3G*, these packages assess whether or not models of neutral evolution can recapitulate the observed molecular data. *A3G* sequences were screened for recombination with GARD and SBR programs in DataMonkey and the data set was partitioned according to breakpoints [64]. Analyses were performed for the full-length alignment as well as for each partition to consider possible effects of recombination. The mixed effects model of evolution (MEME) analysis was performed in DataMonkey to identify individual codons subject to diversifying selection with a p-value threshold of 0.05, as determined by a significant proportion of branches in the tree exhibiting a bias towards non-synonymous variation at these sites [41]. The MEME analysis is recommended for analyses of diversifying selection in host genes because it is sensitive to cases of transient or episodic selection, whereas traditional methods are not [41]. The Codeml program was used to determine whether *A3G* is evolving under positive selection (comparison of models M7 and M8) and to identify the individual residues undergoing selection (Nsites) [40]. Maximum likelihood scores were calculated under each model and significant differences were calculated using the Chi-square test. Bayes Empirical Bayes (BEB) analysis was used to pinpoint residues with a posterior probability >0.95 that dN/dS>1.

### Recombinant HIV-1 proviral plasmids

The following SIV *vif* sequences were synthesized by GenScript (without codon optimization): SIVdeb CM5, SIVmus-1 CM1085, SIVsm E041, SIVcol CGU1, SIVwrc 98CI04, SIVolc 97CI12. HIV-2_ROD9_ *vif* and SIVmac239 *vif* were PCR amplified from the full-length molecular clone [65], [66] and other *vif* genes were previously described in [29]. SIV *vif* sequences were appended with a 5′ Kozac sequence and 5′Mlu1 and 3′ Xba1 restriction sites by PCR and cloned into the HIV-1Δ*vif* molecular clone pLaiΔ*env*Luc2Δ*vif*, generated after Nde1-Stu1 deletion in pLaiΔ*env*Luc2. Epitope-tagged versions of select *vif* isolates (SIVcol *vif*, SIVwrc *vif*, and SIVolc *vif*,) were produced by appending a 3′ 3X-FLAG, and they too were cloned into the HIV-1Δ*vif* molecular clone pLaiΔ*env*Luc2Δ*vif*. The resulting proviral plasmids lack *env*, contain a firefly luciferase gene into *nef*, and encode SIV *vif* in the context of the HIV-1 backbone.

### Single-round viral infectivity assays and Western blot analysis

293T cells were plated in 12-well plates at 2.5×10^5^ cells/mL. The following day, cells were cotransfected with 0.4 µg of A3G expression plasmid of an empty expression plasmid, 0.1 µg of L-VSV-G (vesicular stomatitis virus glycoprotein, for pseudotyping), and 0.6 µg of proviral plasmid in a 100 µL transfection volume with TransIT-LT1 lipid transfection reagent (Mirus Bio). Virus supernatants were harvested at 48 hrs and clarified by centrifugation for 5 min at 1,800 rpm, while transfected cells were lysed with NP-40-doc buffer (1% NP-40, 0.2% sodium deoxycholate, 0.12 M NaCl, 20 mM Tris [pH 8.0], 2.4 mM dithiothreitol (DTT) and protease inhibitor cocktail (Roche)) and pelleted for 5 min at 10,000 rpm. Total protein concentration was quantified by Bradford assay and 20 µg was resolved by 10% SDS-PAGE, transferred to polyvinylidene difluoride (PVDF) membranes, and probed with anti-HA (Santa Cruz Biotechnology) or anti-actin (Sigma) antibodies. Virus in the supernatant was quantified by p24 Gag enzyme-linked immunosorbent assay (Advanced Bioscience Laboratories). Two ng of virus was used to infect supT1 cells plated at 3.8×10^5^ cells/mL in the presence of 20 µg/mL DEAE-Dextran, in a total volume of 100 µL. Virus infections were performed in triplicate for 48 hrs. Luciferase activity was measured with 100 µL of Bright-Glo Luciferase Assay Reagent (Promega).

### Synthesis of chimeric A3G and mutant A3G expression plasmids

Chimeric A3G plasmids Chi A, B, and D were produced between mantled colobus A3G and AGM haplotype I A3G [29] by restriction digest with BamH1, BstX1, and Apa1, respectively. The remaining chimeras were produced by overlap PCR with reaction-specific primer sets. Mutagenesis of colobus A3G and chimeras Chi A and C was performed using the Quikchange II XL Site-Directed Mutagenesis Kit (Agilent Technologies).

### Accession numbers

The GenBank accession numbers for OWM *A3G* sequences produced from this study are KC176173-KC176194.

## Supporting Information

Figure S1 **Phylogenetic reconstruction of OWM** ***A3G*** **.** (A) The phylogeny utilized for evolutionary analyses, modeled after the accepted OWM species phylogeny [37]. (B) A bootstrapped maximum likelihood phylogeny of OWM *A3G* produced using the web-based version of PhyML (phylogeny.fr) and depicted as a cladogram. Note the somewhat different branching topology and paraphyly of some intraspecies *A3G* sequences.
(TIF)

Figure S2 **Protein alignment of OWM A3G.** *A3G* nucleotide sequences from OWM species, including intraspecies variants detected for some species, were aligned in ClustalW and translated to amino acid code. Residues that meet or approach the threshold for diversifying selection, as detected by MEME analysis, are highlighted in green. Gaps removed for evolutionary analysis have been restored, and codon numbering corrected to reflect positioning in AGM A3G.
(PDF)

Figure S3 **SIVolc Vif antagonizes colobus A3G.** (A) Single-round infectivity assays were performed with HIV-1ΔVif and HIV-1 expressing SIV Vif proteins produced in the presence of AGM haplotype I A3G or Colobus A3G. Error bars indicate standard deviation from the mean of three infection replicates. SIVolc Vif, but not Vif from other characterized isolates of SIV that infect *Colobinae* hosts, overcomes restriction by A3G of the mantled colobus (*Colobus guereza*). (B) Anti-HA and anti-FLAG western blot analysis were used to measure expression of A3G and Vif in virus producing cells, respectively. Note that expression of Vif from SIVcol, the species-specific lentivirus of the mantled colobus monkey, was not detected. SIVwrc Vif was expressed, but demonstrated no activity against colobus A3G.
(TIF)

Table S1 **Comparison of results from selection models implemented in Codeml.** A comparison of M7 and M8 models, assuming the F3x4 codon frequency model and an initial ω value of 0.4, was used to identify individual residues of A3G undergoing positive selection. Runs using alternative codon frequency models (F1x4 and codon table), and alternative selection models (M1 and M2) generated similar results. M7 and M8 allow the dN/dS values for each site to vary according to a beta distribution, but M8 allows for dN/dS values greater than 1 (selection). The difference in likelihood scores generated by each model was calculated by likelihood ratio tests, and a chi-square distribution with 2 degrees of freedom was used to assess statistical significance. A comparison of M0 (one ratio) and M1 (free ratio) models was used to distinguish between pervasive selection throughout the tree and episodic selection localized to individual branches. 2(n-1)-1 degrees of freedom were used in these likelihood ratio tests. Tests were run separately for three data sets: OWM+Hominoids, OWM alone, and OWM alone partitioned between recombination breakpoints.
(TIF)

Table S2 **Sites undergoing diversifying selection in OWM** ***A3G*** **.** The web-based version of HyPhy (datamonkey.org) was used to perform the MEME and REL analyses. The Nsites feature in Codeml was used to perform the BEB analysis. Shown are sites identified by MEME to meet or approach the threshold for diversifying selection (p<0.05). REL and BEB scores for these same sites are reported as posterior probabilities and as Bayes factors (in parentheses).
(TIF)
