# Supplementary figures and images for: Convergence and Divergence in the Evolution of the APOBEC3G-Vif Interaction Reveal Ancient Origins of Simian Immunodeficiency Viruses
Source: PLoS Pathog. 2013 Jan 24;9(1):e1003135. doi: 10.1371/journal.ppat.1003135 (PMC3554591; doi:10.1371/journal.ppat.1003135)

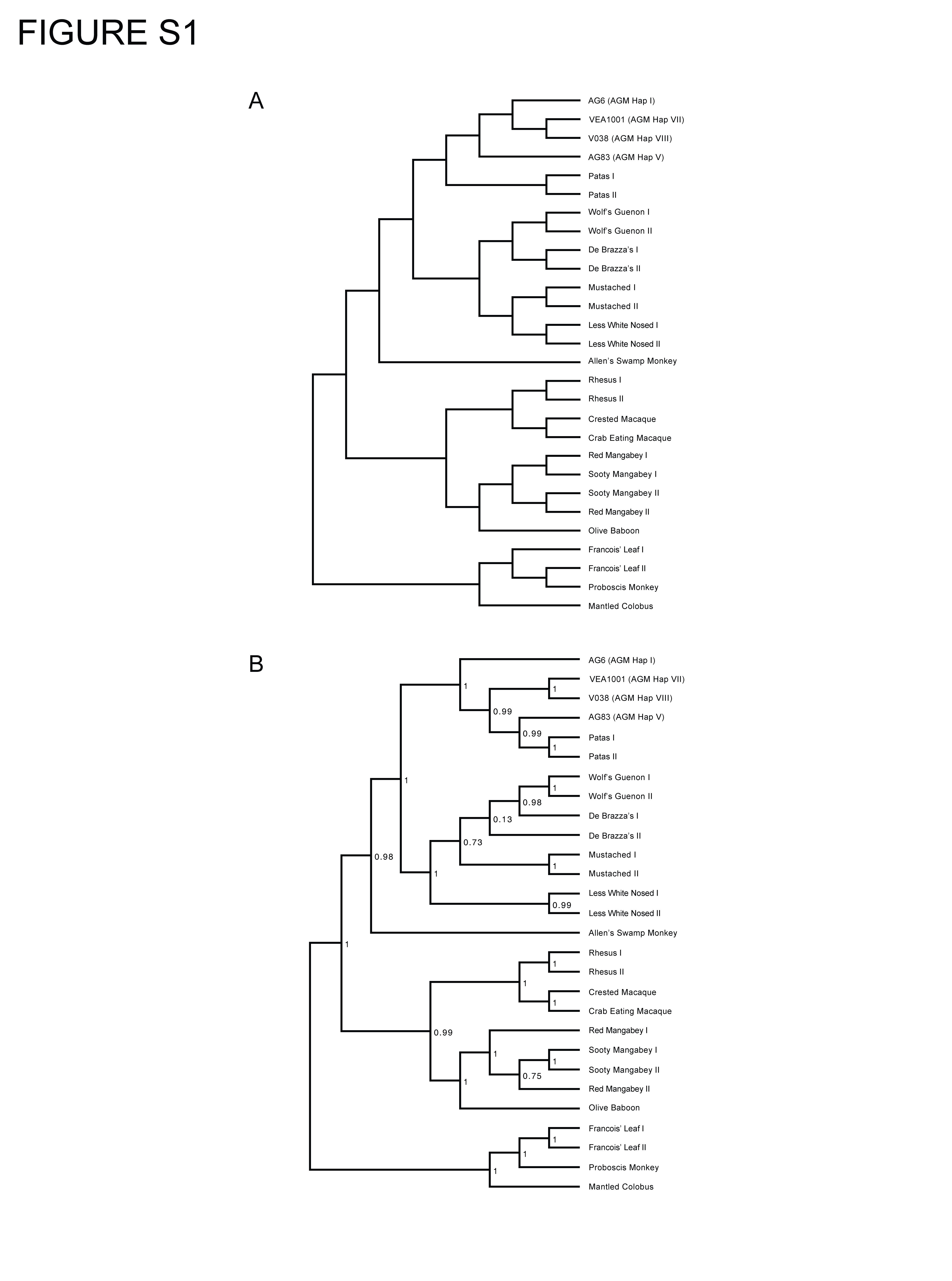

Supplement: Figure S1 — Phylogenetic reconstruction of OWM A3G . (A) The phylogeny utilized for evolutionary analyses, modeled after the accepted OWM species phylogeny [37]. (B) A bootstrapped maximum likelihood phylogeny of OWM A3G produced using the web-based version of PhyML (phylogeny.fr) and depicted as a cladogram. Note the somewhat different branching topology and paraphyly of some intraspecies A3G sequences. (TIF) [file ppat.1003135.s001.tif]

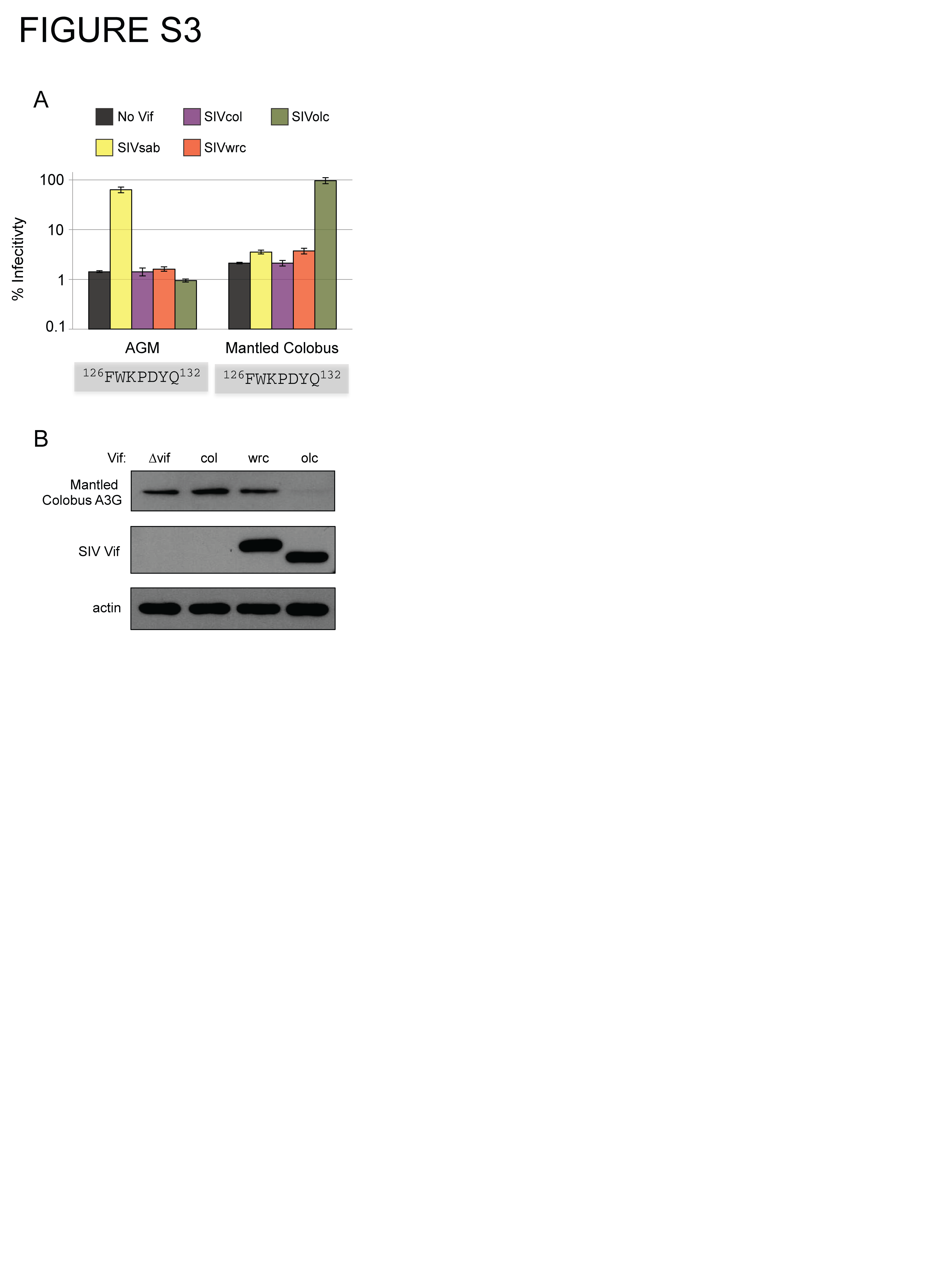

Supplement: Figure S3 — SIVolc Vif antagonizes colobus A3G. (A) Single-round infectivity assays were performed with HIV-1ΔVif and HIV-1 expressing SIV Vif proteins produced in the presence of AGM haplotype I A3G or Colobus A3G. Error bars indicate standard deviation from the mean of three infection replicates. SIVolc Vif, but not Vif from other characterized isolates of SIV that infect Colobinae hosts, overcomes restriction by A3G of the mantled colobus (Colobus guereza). (B) Anti-HA and anti-FLAG western blot analysis were used to measure expression of A3G and Vif in virus producing cells, respectively. Note that expression of Vif from SIVcol, the species-specific lentivirus of the mantled colobus monkey, was not detected. SIVwrc Vif was expressed, but demonstrated no activity against colobus A3G. (TIF) [file ppat.1003135.s003.tif]

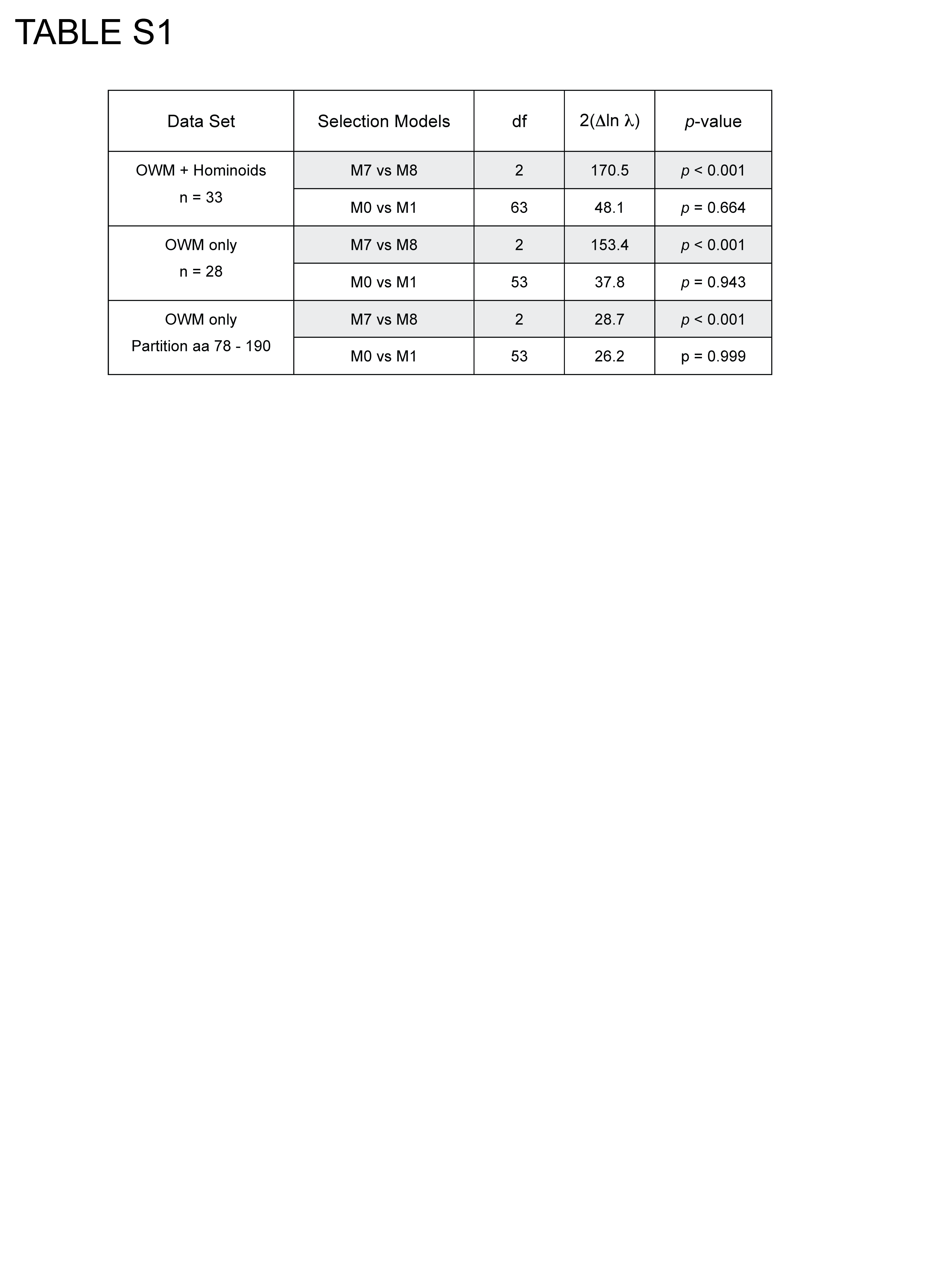

Supplement: Table S1 — Comparison of results from selection models implemented in Codeml. A comparison of M7 and M8 models, assuming the F3x4 codon frequency model and an initial ω value of 0.4, was used to identify individual residues of A3G undergoing positive selection. Runs using alternative codon frequency models (F1x4 and codon table), and alternative selection models (M1 and M2) generated similar results. M7 and M8 allow the dN/dS values for each site to vary according to a beta distribution, but M8 allows for dN/dS values greater than 1 (selection). The difference in likelihood scores generated by each model was calculated by likelihood ratio tests, and a chi-square distribution with 2 degrees of freedom was used to assess statistical significance. A comparison of M0 (one ratio) and M1 (free ratio) models was used to distinguish between pervasive selection throughout the tree and episodic selection localized to individual branches. 2(n-1)-1 degrees of freedom were used in these likelihood ratio tests. Tests were run separately for three data sets: OWM+Hominoids, OWM alone, and OWM alone partitioned between recombination breakpoints. (TIF) [file ppat.1003135.s004.tif]

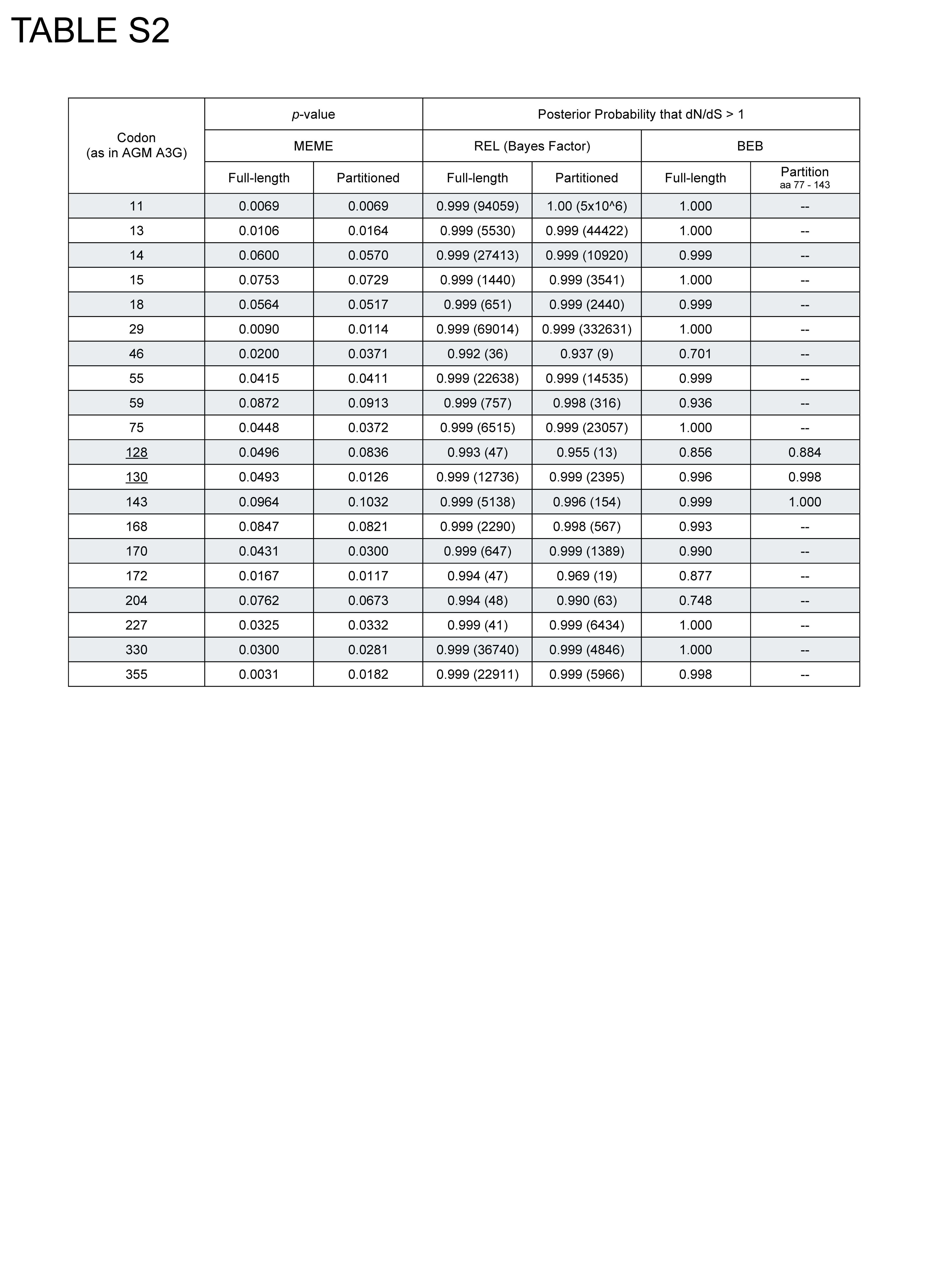

Supplement: Table S2 — Sites undergoing diversifying selection in OWM A3G . The web-based version of HyPhy (datamonkey.org) was used to perform the MEME and REL analyses. The Nsites feature in Codeml was used to perform the BEB analysis. Shown are sites identified by MEME to meet or approach the threshold for diversifying selection (p<0.05). REL and BEB scores for these same sites are reported as posterior probabilities and as Bayes factors (in parentheses). (TIF) [file ppat.1003135.s005.tif]
