# Supplementary material for: Convergence and Divergence in the Evolution of the APOBEC3G-Vif Interaction Reveal Ancient Origins of Simian Immunodeficiency Viruses
Source: PLoS Pathog. 2013 Jan 24;9(1):e1003135. doi: 10.1371/journal.ppat.1003135 (PMC3554591; doi:10.1371/journal.ppat.1003135)
Supplement: Figure S2 — Protein alignment of OWM A3G. A3G nucleotide sequences from OWM species, including intraspecies variants detected for some species, were aligned in ClustalW and translated to amino acid code. Residues that meet or approach the threshold for diversifying selection, as detected by MEME analysis, are highlighted in green. Gaps removed for evolutionary analysis have been restored, and codon numbering corrected to reflect positioning in AGM A3G. (PDF) [file ppat.1003135.s002.pdf]

FIGURE S2

Position markers: 1, 11, 13, 14, 15, 18, 29, 46

AG6_AGM_Hap_I	MKPQIRNMVE	RMKPGIFVYY	FNNRPILSGR	NIVWLCCEVK	TKDPSGPPLD
VEA1001_AGM_Hap_VII	MKPQIRNMVE	RMKPGIFVYY	FNNRPILSGR	NIVWLCCEVK	TKDPSGPPLD
V038_AGM_Hap_VIII	MKPQIRNMVE	RMKPGIFVYY	FNNRPILSGR	NIVWLCCEVK	TKDPSGPPLD
AG83_AGM_Hap_V	MKPQIRNMVE	RMKPGIFVYY	FNNRPILSGR	NTVWLCYEVK	TKDPSGPPLD
Patas_I	MKPQIRNMVE	QMEPDIFVYY	FNNKPILSHR	NTVWLCYEVK	TKDPSGPPLD
Patas_II	MKPQIRNMVE	QMEPDIFVYY	FNNKPILSHR	NTVWLCYEVK	TKDPSGPPLD
Wolf's_Guenon_I	MKPQIRNMVE	RMKRGIFVYY	FNNKPILSDR	NTVWLCCEVK	TKDPSGPPLD
Wolf's_Guenon_II	MKPQIRNMVE	RMKRGIFVYY	FNNKPILSDR	NTVWLCCEVK	TKDPSGPPLD
De_Brazza's_I	MKPQIRNMVK	RMKPGIFVYY	FNNKPILSGR	NTVWLCCEVK	TKDPSGPPLD
De_Brazza's_II	MKPQNRNMVE	RMKPGIFVYY	FNNKPILSGR	NTVWLCCEVK	TKDPSGPPLD
Less_White_Nosed_I	MKPQIRNMVK	RMKPGIFVYY	FNNKPILSGR	NTVWLCCEVK	TKDPSGPPLD
Less_White_Nosed_II	MKPQIRNMVK	RMKPGIFVYY	FNNKPILSGR	NTVWLCCEVK	TKDPSGPPLD
Mustached_I	MKPQIRNMVK	RMKPGIFVYY	FNNKPILSGR	NTVWLCCEVK	TKDPSGPPLD
Mustached_II	MKPQIRNMVK	RMKPGIFVYY	FNNKPILSGR	NTVWLCCEVK	TKDPSGPPLD
Allen's_Swamp	MKPQIRNMVE	QMEPDIFVYY	FNNRPILSGR	NTVWLCCEVK	TKDPSGPPLE
Rhesus_I	MKPQIRNMVE	PMDPRTFVSN	FNNRPILSGL	DTVWLCCEVK	TKDPSGPPLD
Rhesus_II	MKPQIRNMVE	PMDPRTFVSN	FNNRPILSGL	NTVWLCCEVK	TKDPSGPPLD
Crab_Eating_Macaque	MQPQYRNTVE	RMYRGTFFYN	FNNRPILSRR	NTVWLCYEVK	TRGPSMPTWD
Crested_Macaque	MKPQFRNTVE	RMYRGTFFYS	FNNRPILSRR	NTVWLCYEVK	TRGPSMPTWG
Red_Mangabey_II	MKPQIRNMVK	RMKTGIFVSN	FNNKPILSGR	NTVWLCCEVK	TKDPSGPPLD
Red_Mangabey_I	MKPQIRNMVK	RMKTGIFVSN	FNNKPILSGR	NTVWLCCELK	TKDPSGPPLD
Sooty_I	MKPQIRKMVK	QMKTGIFVSN	FNNKPILSGR	NTVWLCCEVK	TKDPSGPPLD
Sooty_II	MKPQIRNMVK	RMKTGIFVSN	FNNKPILSGR	NTVWLCCEVK	TKDPSGPPLD
Olive_Baboon	MKPQIRNMVK	RMKADIFVSN	FNNRPILSGR	NTVWLCCEVN	TKDPSGPPLD
Francois'_Leaf_I	MKPQIRNMVE	VMYPKRFKYF	FNNEPILSRR	NTVWLCYEVK	TKDSSGPPLD
Francois'_Leaf_II	MKPQIRNMVE	VMYPKRFKYF	FNNKPILSRR	NTVWLCYEVK	TKDSSGPPLD
Proboscis	MKPQIRNMVE	VMYPKRFKYF	FNNKPILSHR	NTVWLCYEVK	TKDPSGTLLD
Colobus	MKPQIRNMVE	VMLPKRFGYF	FNNKPILSHR	NTVWLCYEVK	TKDPSGPPLD

Position markers: 55, 59, 75

AG6_AGM_Hap_I	ANIFQGEVYP	KAK...DHPE	MKFLHWFRKW	K.LHRDQEYE	VTWYVSWSPC
VEA1001_AGM_Hap_VII	ANIFQGELYP	EAK...DHPE	MKFLHWFRKW	K.LHRDQEYE	VTWYVSWSPC
V038_AGM_Hap_VIII	ANIFQGELYP	EAK...DHPE	MKFLHWFRKW	K.LHRDQEYE	VTWYVSWSPC
AG83_AGM_Hap_V	ANIFQGELYP	EAK...DHPE	MKFLHWFREW	K.LHRDQEYE	VTWYVSWSPC
Patas_I	ANIFQGELYP	EAK...DHPE	MKFLHWFRKW	K.LHRDQEYE	VTWYVSWSPC
Patas_II	ANIFQGELYP	EAK...DHPE	MKFLHWFRKW	K.LHRDQEYE	VTWYVSWSPC
Wolf's_Guenon_I	AKIFQDEVYS	KPK...DHPE	MRFLHWFRKW	K.LHRDQEYE	VTWYVSWSPC
Wolf's_Guenon_II	AKIFQDEVYS	KPK...DHPE	MRFLHWFRKW	K.LHRDQEYE	VTWYVSWSPC
De_Brazza's_I	AKIFQDEVYS	KPK...DHPE	MRFLHWFRKW	K.LHRDQEYE	VTWYVSWSPC
De_Brazza's_II	TKIFQGEVYS	KAE...DHPE	MSFLRWFHKW	K.LHRDQEYE	VTWYVSWSPC
Less_White_Nosed_I	AKIFQGEVYS	KPK...DHPE	MRFLHWFRKW	K.LHRDQEYE	VTWYVSWSPC
Less_White_Nosed_II	AKIFQGEVYS	KPK...DHPE	MRFLHWFRKW	K.LHRDQEYE	VTWYVSWSPC
Mustached_I	AKIFQGEVYS	KPK...DHPE	MRFLHWFRKW	K.LHRDQEYE	VTWYVSWSPC
Mustached_II	AKIFQGEVYS	KPK...DHPE	MRFLHWFRKW	K.LHRDQEYE	VTWYVSWSPC
Allen's_Swamp	ANIFQGEVYS	KAK...DHPE	MCFLDWFLEW	K.LHRDQEYE	VTWYVSWSPC
Rhesus_I	AKIFQGKVYP	KAK...YHPE	MRFLRWFHKW	K.LHHDQEYK	VTWYVSWSPC
Rhesus_II	AKIFQGKVYP	KAK...YHPE	MRFLQWFREW	K.LHHDQEYK	VTWYVSWSPC
Crab_Eating_Macaque	TKIFRGQVRS	KAK...YHPE	MRFLHWFREW	K.LHHDQEYK	VTWYVSWSPC
Crested_Macaque	TKIFRGQVYS	KAK...YHPE	MRFLRWFSKW	K.LHHDQEYK	VTWYVSWSPC
Red_Mangabey_II	AKIFRGKVYS	KAK...YHPE	MRFLRWFCKW	K.LHRDQEYE	VTWYVSWSPC

55 59 75

Red_Mangabey_I	AKIFRGKVYS	KAK...YHPE	MRFLRWFCKW	K.LHRDQEYE	VTWYVSWSPC
Sooty_I	AKIFRGKVYS	KAK...YHPE	MRFLRWFLKW	K.LHRDQEYE	VTWYVSWSPC
Sooty_II	AKIFPGKVYS	KAK...YHPE	MRFLRWFRKW	K.LHRDQEYE	VTWYVSWSPC
Olive_Baboon	AKIFRGKVYS	KAK...YHPE	MRFLHWFRKW	K.LHRDQEYE	VTWYVSWSPC
Francois'_Leaf_I	ANIFRGQVSA	KDKSCEDHPE	MRFLHWFREW	RQLHRDQEYE	VTWYVSWSPC
Francois'_Leaf_II	ANIFRGQVSA	KDKSCEDHPE	MRFLHWFREW	RQLHRDQEYE	VTWYVSWSPC
Proboscis	ANIFRGQVSA	KDKSCEDHPE	MRFLHWFRKW	K.LHHDQEYE	VTWYVSWSPC
Colobus	ANIFQGQVSF	KDKSCKDHPE	MRFLHWFRKW	K.LHCDQEYE	VTWYVSWSPC

128 130 143

AG6_AGM_Hap_I	TRCANSVATF	LAKDPKVTLT	IFVARLYYFW	KPDYQQALRI	LCQERGGPHA
VEA1001_AGM_Hap_VII	TRCANSVATF	LAKDPKVTLT	IFVARLYYFW	EPDYQQALRI	LCQKRGGPHA
V038_AGM_Hap_VIII	TRCANSVATF	LAEDPKVTLT	IFVARLYYFW	KPHYQEALRI	LCQKRGGPHA
AG83_AGM_Hap_V	TRCANSVATF	LAKDPKVTLT	IFVARLYYFW	KPDYQQALRI	LCQKRGGPHA
Patas_I	TRCANSVATF	LAEDPKVTLT	IFVARLYYFW	KPDYQEALRI	LCQKRGGPHA
Patas_II	TRCANSVATF	LAEDPKVTLT	IFVARLYYFW	KPDYQEALRI	LCQKRGGPHA
Wolf's_Guenon_I	TRCANNVATF	LAKDPKVTLT	IFVARLYYFW	KPAYQEALRI	LCQKRDGPHA
Wolf's_Guenon_II	TRCANNVATF	LAKDPKVTLT	IFVARLYYFW	KPAYQEALRI	LCQKRDGPHA
De_Brazza's_I	TRCANNVATF	LAKDPKVTLT	IFVARLYYFW	KPAYQEALRI	LCQKRDGPHA
De_Brazza's_II	TRCANNVATF	LAEDPKVTLT	IFVARLYYFW	KPAYQEALRI	LCQKRGGPHA
Less_White_Nosed_I	TRCANSVATF	LAEDPKVTLT	IFVARLYYFW	KPDYQEALRI	LCQKRGGPHA
Less_White_Nosed_II	TRCANSVATF	LAKDPKVTLT	IFVARLYYFW	KPAYQEALRI	LCQKRDGPHA
Mustached_I	TRCANNVATF	LAKDPKVTLT	IFVARLYYFW	KPAYQEALRI	LCQKRGGPHA
Mustached_II	TRCANNVATF	LAKDPKVTLT	IFVARLYYFW	EPAYQEAPRI	LCQKRGGPHA
Allen's_Swamp	TRCANSVTTF	LAEDPKVTLT	IFVARLYYFW	KPDYQEALRI	LCQKRGGPHA
Rhesus_I	TRCANSVATF	LAKDPKVTLT	IFVARLYYFW	KPDYQQALRI	LCQKRDGPHA
Rhesus_II	TRCANSVATF	LAKDPKVTLT	IFVARLYYFW	KPNYQQALRI	LCQKRDGPHA
Crab_Eating_Macaque	TRCANSVATF	LAKDPKVTLT	IFVARLYYFW	KPDYQQALRI	LCQKRGGLHA
Crested_Macaque	TRCANSVATF	LAKDPKVTLT	IFVARLYYFW	KPDYQQALRI	LCQKRGGPHA
Red_Mangabey_II	TGCANSVATF	LAEDPKVTLT	IFVARLYYFW	KPDYQEALRV	LCQKRGSPHA
Red_Mangabey_I	TGCANSVATF	LAEDPKVTLT	IFVARLYYFW	KPDYQEALRV	LCQKRGSPHA
Sooty_I	TGCASSVATF	LAKDPKVTLT	IFVARLYYFW	KPDYQEALRV	LCQKRGSPHA
Sooty_II	TGCANSVATF	LAEDPKVTLT	IFVARLYYFW	KPDYQEALRV	LCQKRGSPHA
Olive_Baboon	TGCANSVATF	LAEDPKVTLT	IFVARLYYFW	KPDYQEALRV	LCQKRGSPHA
Francois'_Leaf_I	ARCANSVATF	LAEDPKVTLT	IFVARLYFFW	KPDYQKALRS	LCQKRDGPHA
Francois'_Leaf_II	ARCANSVATF	LAEDPKVTLT	IFVARLYFFW	KPDYQKALRS	LCQKRDGPHA
Proboscis	ARCANSVATF	LVEDPKVTLT	IFVARLYFFW	KPDYQEALRN	LCQKRDGPHA
Colobus	ARCANSVATF	LAEDPKVTLT	IFVARLYFFW	KPDYQEALRN	LCQKRGGAHA

168 170 172

AG6_AGM_Hap_I	TMKIMNYSEF	QHCWNEFVDG	QGKPFKPRKN	LPKHYTLLHA	TLGELLRHVM
VEA1001_AGM_Hap_VII	TMKIMNYNEF	QHCWNEFVDG	QGKPFKPRKN	LPKHYTLLHA	TLGELLRHVM
V038_AGM_Hap_VIII	TMKIMNYNEF	QHCWNEFVDG	QGKPFKPRKN	LPKHYTLLHA	TLGELLRHVM
AG83_AGM_Hap_V	TMKIMNYNEF	QHCWNKFVDG	QGKPFKPRKN	LPKHYTLLHA	TLGELLRHVM
Patas_I	TMKIMNYNEF	QHCWNEFVDS	QGKPFKPRKN	LPKHYTLLHA	TLGELLRHVM
Patas_II	TMKIMNYNEF	QHCWNEFVDS	QGKPFKPRKN	LPKHYTLLHA	TLGELLRHVM
Wolf's_Guenon_I	TMKIMNYNEF	QHCWNEFVDG	QGKPFKPRKN	LPKHYTLLHA	TLGELLRHVM
Wolf's_Guenon_II	TMKIMNYNEF	QHCWNEFVDG	QGKPFKPRKN	LPKHYTLLHA	TLGELLRHVM
De_Brazza's_I	TMKIMNYNEF	QHCWNEFVDG	QGKPFKPRKN	LPKHYTLLHA	TLGELLRHVM
De_Brazza's_II	TMKIMNYNEF	QHCWNEFVDG	QGKSFKPRKN	LPKHYTLLHA	TLGELLRHVM
Less_White_Nosed_I	TMKIMNYNEF	QHCWNEFVDG	QGVPFKPRKN	LPKHYTLLHA	TLGELLRRVM
Less_White_Nosed_II	TMKIMNYNEF	QHCWNEFVDG	QGKPFKPRKN	LPKHFTLLHA	TLGELLRRVM
Mustached_I	TMKIMNYDEF	QHCWNEFVDG	QGMPFKPRKN	LPKHYTLLHA	TLGELLRHVM
Mustached_II	TMKIMNYDEF	QHCWNEFVDG	QGMPFKPRKN	LPKHYTLLHA	TLGELLRHVM

```
                                                168 170 172
Allen's_Swamp            TMKIMNYNEF QHCWNKFVDG QGKPRKPRKN PPKHYTLLHA TLGELLRHLM
Rhesus_I                 TMKIMNYNEF QDCWNKFVDG RGKPFKPWNN LPKHYTLLQA TLGELLRHLM
Rhesus_II                TMKIMNYNEF QDCWNKFVDG RGKPFKPWNN LPKHYTLLQA TLGELLRHLM
Crab_Eating_Macaque      TMKIMNYNEF QDCWNKFVDG GGKPFKPRNN LPKHYTLLQA TLGELLRHLM
Crested_Macaque          TMKIMNYNEF QDCWNKFVDG RGKPFKPRNN LPKHYTLLQA TLGELLRHLM
Red_Mangabey_II          TMKIMNYNEF QHCWNKFVRG RREPFEPWEN LPKHYTLLHA TLGELLRHLM
Red_Mangabey_I           TMKIMNYNEY QHCWNKFVRG RREPFEPWEN LPKHYTLLHA TLGELLRHLM
Sooty_I                  TMKIMNYNEF QHCWNKFVRG RREPFEPWEN LPKHYTLLHA TLGELLRHLM
Sooty_II                 TMKIMNYNEF QHCWNKFVRG RREPFEPWEN LPKHYTLLHA TLGELLRHLM
Olive_Baboon             TMKIMNYNEF QHCWNKFVRG RREPFEPWEN LPKHYTLLHA TLGELLRHLM
Francois'_Leaf_I         TMKIMNYDEF QHCWDKFVYR PKKPFKPRKN LPKHYTLLHT MLGELLRHLM
Francois'_Leaf_II        TMKIMNYDEF QHCWDKFVYR PKKPFKPRKN LPKHYTLLHT MLGELLRHLM
Proboscis                TMKIMNYDEF QHCWNNFVHR PKKPFKPREN LPKHYTLLHT LLGELLRHLM
Colobus                  TMKIMNYDEF QHCWSKFVYR PEKPFKPWKN LPKHYTLLHN ILGELLRHLM

                                204                   227
AG6_AGM_Hap_I            DPGTFTSNFN NKPWVSGQRE TYLCYKVERS HNDTWVLLNQ HRGFLRNQAP
VEA1001_AGM_Hap_VII      DPGTFTSNFN NKPWVSGQRE TYLCYKVERS HNDTWVLLNQ HRGFLRNQAP
V038_AGM_Hap_VIII        DPGTFTSNFN NKPWVSGQRE TYLCYKVERS HNDTWVLLNQ HRGFLRNQAP
AG83_AGM_Hap_V           DPGTFTSNFN NKPWVSGQRE TYLCYKVERL HNDTWVLLNQ HRGFLRNQAP
Patas_I                  DPGTFTSNFN NKPWVSGQRE TYLCYKVERS HNDTWVLLNQ HRGFLRNQAP
Patas_II                 DPGTFTSNFN NKPWVSGQRE TYLCYKVERS HNDTWVLLNQ HRGFLRNQAP
Wolf's_Guenon_I          DPGTFTSNFN NKPWVSGQRE TYLCYKVERS HNDTWVLLNQ HRGFLRNQAP
Wolf's_Guenon_II         DPGTFTSNFN NKPWVSGQRE TYLCYKVERS HNDTWVLLNQ HRGFLRNQAP
De_Brazza's_I            DPGTFTSNFN NKPWVSGQRE TYLCYKVERS HNDTWVLLNQ HRGFLRNQAP
De_Brazza's_II           DPGTFTSNFN NKPWVSGQRE TYLCYKVERS HNDTWVLLNQ HRGFLRNQAP
Less_White_Nosed_I       DPGTFTSNFN NKPWVSGQRE TYLCYKVERS HNDTWVLLNQ HRGFLRNQAP
Less_White_Nosed_II      DPGTFTSNFN NKPWVSGQRE TYLCYKVERL HNDTWVLLNQ HRGFLRNQAP
Mustached_I              DPDTFTSNFN NKPWVSGQRE TYLCYKVERS HNDTWVLLNQ HRGFLRNQAP
Mustached_II             DPDTFTSNFN NKPWVSGQRE TYLCYKVERS HNDTWVLLNQ HRGFLRNQAP
Allen's_Swamp            DPGTFTSNFN NTPWVGGQHE TYLCYKVERW HNDTWVLLNQ HRGFLHNQAP
Rhesus_I                 DPGTFTSNFN NKPWVSGQHE TYLCYKVERL HNDTWVPLNQ HRGFLRNQAP
Rhesus_II                DPGTFTSNFN NKPWVSGQHE TYLCYKVERL HNDTWVPLNQ HRGFLRNQAP
Crab_Eating_Macaque      DPGTFTSNFN NKPWVSGQHE TYLCYKVERL HNDTWVPLNQ HRGFLRNQAP
Crested_Macaque          DPGTFTSNFN NKPWVSGQHE TYLCYKVERL HNDTWVPLNQ HRGFLRNQAP
Red_Mangabey_II          DPGTFTSNFN NKLWVSGQHE TYLCYKVERP HNDTWVLLNR HRGFLQNQAP
Red_Mangabey_I           DPGTFTSNFN NKLWVSGQHE TYLCYKVERP HNDTWVLLNQ HRGFLQNQAP
Sooty_I                  DPGTFTSNFN NKLWVSGQHE TYLCYKVERP HNDTWVLLNQ HRGFLQNQAP
Sooty_II                 DPGTFTSNFN NKLWVSGQHE TYLCYKVERP HNDTWVLLNQ HRGFLQNQAP
Olive_Baboon             DPGTFTSNFY NKPWVSGQHE TYLCYKVERL HNGTWVPLNQ HRGFLRNQAP
Francois'_Leaf_I         DPDTFTLNFN NEPWVSGQHE SYLCYKVECL DNGTWVPMDE HRGFLRNQAP
Francois'_Leaf_II        DPDTFTLNFN NEPWVSGQHE SYLCYKVECL DNGTWVPMDE HRGFLRNQAP
Proboscis                DPGTFTLNFN NEPWVSGQHE SYLCYKVERL DNGTWVPMDE HRGFLRNQAP
Colobus                  DPGTFTLNFN NEPWVSGQHE TYLCYKVERL DNGTWVPMDE HRGFLCNQAP

AG6_AGM_Hap_I            DRHGFPKGRH AELCFLDLIP FWKLDDQQYR VTCFTSWSPC FSCAQKMAKF
VEA1001_AGM_Hap_VII      DRHGFPKGRH AELCFLDLIP FWKLDDQQYR VTCFTSWSPC FSCAQKMAKF
V038_AGM_Hap_VIII        DRHGFPKGRH AELCFLDLIP FWKLDDQQYR VTCFTSWSPC FSCAQKMAKF
AG83_AGM_Hap_V           DRHGFPKGRH AELCFLDLIP FWKLDDQQYR VTCFTSWSPC FSCAQKMAKF
Patas_I                  DRHGFPKGRH AELCFLDLIP FWKLDDQQYR VTCFTSWSPC LSCAQKMAKF
Patas_II                 DRHGFPKGRH AELCFLDLIP FWKLDDQQYR VTCFTSWSPC FSCAQKMAKF
Wolf's_Guenon_I          DRHGFPKGRH AELCFLDLIP FWKLDDQQYR ITCFTSWSPC FSCAQKTAKF
Wolf's_Guenon_II         DRHGFPKGRH AELCFLDLIP FWKLDDQQYR ITCFTSWSPC FSCAQKMAKF
```

```
De_Brazza's_I           GRHGFPKGRH AELCFLDLIP FWKLDDQQYR ITCFTSWSPC FSCAQKMAKF
De_Brazza's_II          DRHGFPKGRH AELCFLDLIP FWKLDDQQYR ITCFTSWSPC FSCAQKMAKF
Less_White_Nosed_I      DRHGFPKGRH AELCFLDLIP FWKLDDQQYR ITCFTSWSPC FSCAQKMAKF
Less_White_Nosed_II     DRHGFPKGRH AELCFLDLIP FWKLDDQQYR ITCFTSWSPC SSCAQEMAKF
Mustached_I             DRHGFPKGRH AELCFLDLIP FWKLDDQQYR VTCFTSWSPC FSCAQKMAKF
Mustached_II            DRHGFPKGRH AELCFLDLIP FWKLDDQQYR VTCFTSWSPC FSCAQKMAKF
Allen's_Swamp           DRHGFPKGRH AELCFLDLIH FWKLDGQQYR VTCFTSWSPC FSCAQKMAKF
Rhesus_I                NIHGFPKGRH AELCFLDLIP FWKLDGQQYR VTCFTSWSPC FSCAQEMAKF
Rhesus_II               NIHGFPKGRH AELCFLDLIP FWKLDGQQYR VTCFTSWSPC FSCAQEMAKF
Crab_Eating_Macaque     NIHGFPKGRH AELCFLDLIP FWKLDGQQYR VTCFTSWSPC FSCAQEMAKF
Crested_Macaque         NIHGFPKGRH AELCFLDLIP FWKLDGQQYR VTCFTSWSPC FSCAQEMAKF
Red_Mangabey_II         DIHGFPKGRH AELCFLDLIP FWKLDGQQYR VTCFTSWSPC FNCAQEMAKF
Red_Mangabey_I          DIHGFPKGRH AELCFLDLIP LWKLDGQQYR VTCFTSWSPC FNCAQEMAKF
Sooty_I                 DIHGFPKGRH AELCFLDLIP LWKLDGQQYR VTCFTSWSPC FNCAQEMAKF
Sooty_II                DIHGFPKGRH AELCFLDLIP LWKLDGQQYR VTCFTSWSPC FNCAQEMAKF
Olive_Baboon            DIHGFPKGRH AELCFLDLIP FWKLDGQQYR VTCFTSWSPC FSCAQEMAKF
Francois'_Leaf_I        NKHGFPKGRH AELCFLDLIP FWKLDGQQYR VTCFTSWSPC FSCAQEMATF
Francois'_Leaf_II       NKHGFPKGRH AELCFLDLIP FWKLDGQQYR VTCFTSWSPC FSCAQEMATF
Proboscis               NKHGFPKGRH AELCFLDLIP FWKLDGQQYR VTCFTSWSPC FSCAQEMAKF
Colobus                 NIHGFPKGRH AELCFLDLIS FWKLDGQQYR VTCFTSWSPC FSCAQEMATF

                                                            330
AG6_AGM_Hap_I           ISNNKHVSLC IFAARIYDDQ GRCQEGLRTL HRDGAKIAVM NYSEFEYCWD
VEA1001_AGM_Hap_VII     ISNNKHVSLC IFAARIYDDQ GRCQEGLRTL HRDGAKIAVM NYSEFEYCWD
V038_AGM_Hap_VIII       ISNNKHVSLC IFAARIYDDQ GRCQEGLRTL HRDGAKIAVM NYSEFEYCWD
AG83_AGM_Hap_V          ISNNKHVSLC IFAARIYDDQ GRCQEGLRTL HRDGAKIAVM NYSEFEYCWD
Patas_I                 ISKKKHVSLC IFAARIYDDQ GRCQEGLRTL HRDGAKIAVM NYSEFEYCWD
Patas_II                ISNNKHVSLC IFAARIYDDQ GRCQEGLRTL HRDGAKIAVM NYSEFEYCWD
Wolf's_Guenon_I         ISNNKHMSLC IFAARIYDDQ GRCQEGLRTL HRVGAKIAMM NYSEFEYCWD
Wolf's_Guenon_II        ISNNKHVSLC IFATRIYDDQ GRCQEGLRTL HRVGAKIAMM NYSEFEYCWD
De_Brazza's_I           ISNNEHVSLC IFAARIYDDQ GRCQEGLRTL HRGGAKIAMM NYSEFEYCWD
De_Brazza's_II          ISNNEHVSLC IFAARIYDDQ GRCQEGLRTL HRGGAKIAMM NYSEFEYCWD
Less_White_Nosed_I      ISDNKHVSLC IFATRIYDDQ GRCQEGLRTL HRGGAKIAMM NYSEFEYCWD
Less_White_Nosed_II     ISDNKHVSLC IFAARIYDDQ GRCQEGLRTL HRDGAKIAMM NYSEFEYCWD
Mustached_I             ISNYKHVSLC IFAARIYDDQ GRCQEGLRTL HRGGAKIAMM NYSEFEYCWD
Mustached_II            ISNYKHVSLC IFAARIYDDQ GRCQEGLRTL HRGGAKIAMM NYSEFEYCWD
Allen's_Swamp           ISNKKHVSLC IFAARIYDDQ GRCQEGLRTL HRVGAKIAMM NYSEFKHCWD
Rhesus_I                ISNNEHVSLC IFAARIYDDQ GRYQEGLRTL HRDGAKIAMM NYSEFEYCWD
Rhesus_II               ISNNEHVSLC IFAARIYDDQ GRYQEGLRTL HRDGAKIAMM NYSEFEYCWD
Crab_Eating_Macaque     ISNNEHLSLC IFAARIYDDQ GRYQEGLRTL HRDGAKIAMM NYSEFKHCWD
Crested_Macaque         ISNNEHVSLC IFAARIYDDQ GRYQEGLRTL HRDGAKIAMM NYSEFEYCWD
Red_Mangabey_II         ISNNKHVSLR IFAARIYDDQ GRCQEGLRTL HRDGAKIAMM NYSEFEYCWD
Red_Mangabey_I          ISNNKHVSLC IFAARIYDDQ GRCQEGLRTL HRDGAKIAMM NYSEFEYCWD
Sooty_I                 ISNNKHVSLR IFAARIYDDQ GRCQEGLRTL HRDGAKIAMM NYSELEYCWD
Sooty_II                ISNNKHVSLR IFAARIYDDQ GRCQEGLRTL HRDGAKIAMM NYSELEYCWD
Olive_Baboon            ISNNEHVSLC IFAARIYDDQ GRCQEGLRTL HRDGAKIAMM NYSEFEYCWD
Francois'_Leaf_I        ISNNKHVTLC IFAARIYDDQ GRGQEGLRAL HTAGAEIAMM NYSEFKHCWD
Francois'_Leaf_II       ISNNKHVTLC IFAARIYDDQ GRGQEGLRAL HTAGAEIAMM NYSEFKHCWD
Proboscis               ISSNKHVSLR IFAARIYDDQ GRGQEGLRAL HTAGAEIAMM NYSEFKHCWD
Colobus                 ISSKKHVSLR IFTARIYDDQ GRCQEGLRTL HTVGAKIAMM NYSEFEYCWD

                               355
AG6_AGM_Hap_I           TFVDRQGRPF QPWDGLDEHS QALSGRLRAI LQNQGN
VEA1001_AGM_Hap_VII     TFVDRQGRPF QPWDGLDEHS QALSGRLRAI LQNQGN
```

```
                                       355                            384
V038_AGM_Hap_VIII         TFVDRQGRPF QPWDGLDEHS QALSGRLRAI LQNQGN
AG83_AGM_Hap_V            TFVDRQGRPF QPWDGLDEHS QALSGRLRAI LQNQGN
Patas_I                   TFVDRQGRPF QPWDGLDEHS QALSGRLRAI LQNQGN
Patas_II                  TFVDRQGRPF QPWDGLDEHS QALSGRLRAI LQNQGN
Wolf's_Guenon_I           TFVDRQGRPF QPWDGLDEHS QALSGRLRAI LQNQGN
Wolf's_Guenon_II          TFVDRQGRPF QPWDGLDEHS QALSGRLRAI LQNQGN
De_Brazza's_I             TFVDRQGHPF QPWDGLDEHS QALSGRLQAI LQNQGN
De_Brazza's_II            TFVDRQGHPF QPWDGLDEHS QALSGRLQAI LQNQGN
Less_White_Nosed_I        TFVDHQGRPF QPWDGLDEHS QALSGRLRAI LQNQGN
Less_White_Nosed_II       TFVDHQGRPF QPWDGLDEHS QALSGRLRAI LQNQGN
Mustached_I               TFVDRQGRPF QPWDGLDEHS QDLSGRLWAI LQNQGN
Mustached_II              TFVDRQGRPF QPWDGLDEHS QDLSGRLWAI LQNQGN
Allen's_Swamp             TFVDRQGRPF QPWDGLDEHS QALSGRLRAI LQNQGN
Rhesus_I                  TFVDRQGRPF QPWDGLDEHS QALSGRLRAI LQNQGN
Rhesus_II                 TFVDCQGCPF QPWDGLDEHS QALSERLRAI LQNQGN
Crab_Eating_Macaque       TFVDRQGRPF QPWDGLDEHS QALSERLRAI LQNQGN
Crested_Macaque           TFVDRQGRPF QPWDGLDEHS QALSERLRAI LQNQGN
Red_Mangabey_II           TFVDRQGRPF QPWDGLDEHS QDLSERLRAI LQNQGN
Red_Mangabey_I            TFVDRQGRPF QPWDGLDEHS QDLSERLRAI LQNQGN
Sooty_I                   TFVDRQGYPF QPWDGLDEHS QALSERLRAT LQNQGN
Sooty_II                  TFVDRQGYPF QPWDGLDEHS QALSERLRAT LQNQGN
Olive_Baboon              TFVDRQGRPF QPWDGLDEHS QDLSGRLRAI LQNQGN
Francois'_Leaf_I          TFVDRQGRPF QPWDGLDEHS QALSGRLRAI LQNQGN
Francois'_Leaf_II         TFVDRQGRPF QPWDGLDEHS QALSGRLRAI LQNQGN
Proboscis                 TFVDRQGRPF QPWDGLDEHS QALSGRLRAI LQNQGN
Colobus                   TFVDHQGRPF QPWDGLDEHS QALSGRLRAI LQNQGN
```
